# A robust Bayesian test for identifying context effects in multiattribute decision-making

**DOI:** 10.3758/s13423-022-02157-2

**Published:** 2022-09-27

**Authors:** Dimitris Katsimpokis, Laura Fontanesi, Jörg Rieskamp

**Affiliations:** Department of Psychology, University of Basel, Missionsstrasse 62A, 4055 Basel, Switzerland

**Keywords:** Context effects, Bayesian models, Attraction effect, Similarity effect, Compromise effect

## Abstract

Research on multiattribute decision-making has repeatedly shown that people’s preferences for options depend on the set of other options they are presented with, that is, the choice context. As a result, recent years have seen the development of a number of psychological theories explaining context effects. However, much less attention has been given to the statistical analyses of context effects. Traditionally, context effects are measured as a change in preference for a target option across two different choice sets (the so-called relative choice share of the target, or RST). We first show that the frequently used definition of the RST measure has some weaknesses and should be replaced by a more appropriate definition that we provide. We then show through a large-scale simulation that the RST measure as previously defined can lead to biased inferences. As an alternative, we suggest a Bayesian approach to estimating an accurate RST measure that is robust to various circumstances. We applied the two approaches to the data of five published studies (total participants, *N* = 738), some of which used the biased approach. Additionally, we introduce the absolute choice share of the target (or AST) as the appropriate measure for the attraction effect. Our approach is an example of evaluating and proposing proper statistical tests for axiomatic principles of decision-making. After applying the AST and the robust RST to published studies, we found qualitatively different results in at least one-fourth of the cases. These results highlight the importance of utilizing robust statistical tests as a foundation for the development of new psychological theories.

**Supplementary Information:**

The online version contains supplementary material available at 10.3758/s13423-022-02157-2https://doi.org/10.3758/s13423-022-02157-2.

Axiomatic principles of decision-making have been at the forefront of psychological research over the last five decades, as a large body of work has questioned their empirical basis. However, past research has shown that it is crucial to use proper statistical methods to analyze the results of test of axiomatic principles. For instance, Regenwetter, Dana, and Davis-Stober (2011) found little evidence against the choice principle of transitivity when reanalyzing past published results with proper methods. The goal of the present study is to identify the methodological pitfalls of a frequently used measure of context effects and to propose new and more robust statistical alternatives to identifying context effects.

Past empirical research has shown that people’s preference for one target option depends on the choice set in which it is presented (e.g., Debreu, 1960; Tversky, 1972; Tversky & Russo, 1969; Rumelhart & Greeno, 1971; Busemeyer, Gluth, Rieskamp, & Turner, 2019; Simonson & Tversky, 1992; Tversky & Simonson, 1993; Roe, Busemeyer, & Townsend, 2001; Huber, Payne, & Puto, 1982; Trueblood, Brown, Heathcote, & Busemeyer, 2013; Dhar & Simonson, 2003; Mishra, Umesh, & Stem, 1993; O’Curry & Pitts, 1995; Wedell, 1991; Choplin & Hummel, 2005). Here, we consider three of the most studied context effects: (1) the *similarity effect*, which is the finding that people prefer a target option when it is presented in a choice set with two other dissimilar options compared to when it is presented in a set with one similar and one dissimilar option (Tversky, 1972); (2) the *attraction effect*, which is the finding that people prefer a target option when it is presented in a choice set with a similar but inferior option (Huber et al., 1982); and (3) the *compromise effect*, which is the finding that people prefer a target option when it is in between two more extreme options in the attribute space (Simonson, 1989). Crucially, these findings violate the independence from irrelevant alternatives (IIA) principle (Luce, 1959), which assumes that the relative preference for two options should not be affected by the presence of other available options (for an overview, see Rieskamp, Busemeyer, & Mellers, 2006).

These context effects have been observed across different domains and tasks: from perceptual decisions (e.g., what is the largest stimulus? e.g., Trueblood et al., 2013; Choplin & Hummel, 2005) to likelihood judgments (e.g., how likely is a runner to win a race? e.g., Windschitl & Chambers, 2004) to preferential decisions (e.g., which consumer product is most preferable? Amir & Levav, 2008; Wedell & Pettibone, 1996; O’Curry & Pitts, 1995; Mishra et al., 1993; Farmer, Warren, El-Deredy, & Howes, 2017; Tversky, 1972; Simonson & Tversky, 1992) to decisions under risk (Mohr, Heekeren, & Rieskamp, 2017; for reviews on context effects see, e.g., Busemeyer, Barkan, Mehta, & Chaturvedi, 2007; Busemeyer et al., 2019; Bettman, Luce, & Payne, 1998; Heath & Chatterjee, 1995; Neumann, Bckenholt, & Sinha, 2016). Moreover, a number of cognitive theories have been developed to explain how and why context effects arise (e.g., Tversky, 1972; Simonson & Tversky, 1992; Wedell, 1991; Tversky & Simonson, 1993; Roe et al., 2001; Usher & McClelland, 2004; Bhatia, 2013; Trueblood, Brown, & Heathcote, 2014; Wollschläger & Diederich, 2012; Noguchi & Stewart, 2018; Soltani, Martino, & Camerer, 2012; Louie, Khaw, & Glimcher, 2013; Howes, Warren, Farmer, El-Deredy, & Lewis, 2016; Spektor, Gluth, Fontanesi, & Rieskamp, 2019; for a recent review, see Wollschlaeger & Diederich, 2020; for systematic comparisons of models see Evans, Holmes, & Trueblood, 2019; Hotaling & Rieskamp, 2018; Turner, Schley, Muller, & Tsetsos, 2018).

Despite the large effort to explain context effects through the development of cognitive models, there has been relatively little effort to develop a statistically sound approach for testing whether the effects exist in the first place. Past work has already shown the challenges of a robust statistical analysis for context effects. For example, Hutchinson, Kamakura, and Lynch (2000) showed that context effects may arise from latent classes of participants who have strong attribute preferences but do not exhibit context effects within each participant class: Context effects can emerge on the aggregate level because of the different latent choice patterns of participants, which can remain unaccounted for by popular statistical tests. Liew, Howe, and Little (2016) provided further evidence for different latent classes of participants in context effects with a Bayesian clustering method. These studies thus suggest that looking only at the aggregate descriptions of the data can provide a misleading picture of context effects.

We propose a Bayesian approach to identifying context effects. Via simulations, we show that our approach is resistant to biases due to different numbers of observations per choice set, in contrast to a frequently used alternative approach. In addition, we reanalyze the data of five published experiments, showing that with our proposed method, the evidence for the existence of context effects partly differs from that reported in the original publications.

## The decision problem

In traditional context-effect experiments, a pair of similar options (say options A and B) is embedded in two different choice sets (i.e., the choice contexts). In some studies, participants initially express their preferences for options A and B when presented as pairs, which is considered a baseline condition, and later the same options are embedded in triplets (e.g., Tversky, 1972; Malkoc, Hedgcock, & Hoeffler, 2013; Mishra et al., 1993; Huber et al., 1982; Simonson & Tversky, 1992; Dhar & Simonson, 2003; Wedell, 1991 among others).

In contrast to the traditional context-effect experiments, we focus on studies that use two triplets to measure context effects, an approach that has been used more often in recent work (e.g., Berkowitsch, Scheibehenne, & Rieskamp, 2014; Trueblood et al., 2013; Trueblood, Brown, & Heathcote, 2015; Farmer et al., 2017; Trueblood et al., 2014, among others). The use of two triplets provides the advantage that any effects of the contexts cannot be confounded with the number of options presented. In experiments with two triplets, two core stimuli (*A* and *B*) are embedded in two different choice sets consisting of three options each (i.e., {*A*,*B*,*C*} and {*A*,*B*,*D*}), whereby only the attribute values of the third option (*C* or *D*) change across contexts. To illustrate the point, consider two cars that trade off on two attributes (see Table 1): Car *A* is highly fuel efficient but is expensive, whereas car *B* is cheaper but less fuel efficient. These two baseline options are embedded in two triplets: In one triplet, car *C* is more fuel efficient than car *A* and more expensive (Set 1), and in the other triplet, car *D* is less fuel efficient than car *B* but is also less expensive (Set 2). This is an example of how one can elicit the compromise effect, according to which adding extreme options in the choice set makes average options seem like compromises: The relative preference for car *A* compared to *B* should increase in the first described set and decrease in the second set. Therefore, car *A* is the *target* option and car *B* is the *competitor* option with respect to the compromise effect in the first set, whereas car *B* is the target and car *A* is the competitor with respect to the compromise effect in the second set.

**Table 1:** Example of a multiattribute choice situation representing the compromise effect

Set	Car	Price (USD)	Fuel efficiency (mpg)
1	*A*	25,000	35
	*B*	20,000	30
	*C*	30,000	40
2	*A*	25,000	35
	*B*	20,000	30
	*D*	15,000	25

The core options *A* and *B* are embedded into two different choice sets. The compromise effect targets option *A* in Set 1 and option *B* in Set 2. Option *A* is the target and option *B* is the competitor in Set 1, and vice-versa in Set 2. USD = U.S. dollars; mpg = miles per gallon

Wedell (1991) introduced the two-triplet paradigm as a means to increase statistical power to detect the attraction effect, since the third option affects the two core stimuli (*A* and *B*) differently in the two choice sets (therefore, providing two opportunities for the emergence of the effect). A recent meta-analysis on the compromise effect confirmed that the two-triplet paradigm elicits the effect more strongly than the one-pair-one-triplet paradigm (Neumann et al., 2016). However, in analyzing the attraction effect, Wedell (1991) used ANOVAs on proportions, where the assumption of homogeneity of variance is by default violated (cf. Jaeger, 2008), and therefore his analysis was not optimal. Since then, the question of what is a robust methodological approach to test context effects has not been raised. We suggest an answer to this question by validating and introducing a Bayesian approach to estimating context effects.

## The relative choice share of the target

To measure the effect of context, we first determine the choice frequency of the target in the first context C_1_ (i.e., *n*_t,C1_) and divide it by the choice frequency of the competitor and the target in the same context (i.e., *n*_t,C1_ + *n*_c,C1_), a measure called the *relative choice share of the target* (RST; cf. Berkowitsch et al., 2014). The RST for one context/triplet will deviate from .50 when either the target or competitor is preferred by the decision-maker. In a second step, the RST is determined for the second context C_2_ as well, that is, *n*_t,C2_ relative to *n*_t,C2_ + *n*_c,C2_. In the second context the options representing the target and the competitor switch their roles. Therefore, in the absence of a context effect, the average RST across both conditions is equal to .50. If the total frequencies with which the target and competitor are chosen across both context are identical (i.e., if *n*_*t*,*C*1_ + *n*_c,C1_ = *n*_t,C2_ + *n*_c,C2_), the RST can be determined as

1 $$RST_{\;\text{UW}} = \frac{n_{\mathrm{t},\mathrm{C}1} + n_{\mathrm{t},\mathrm{C}2}}{n_{\mathrm{t},\mathrm{C}1} + n_{\mathrm{t},\mathrm{C}2} + n_{\mathrm{c},\mathrm{C}1} + n_{\mathrm{c},\mathrm{C}2}},$$

following the definitions of (Berkowitsch et al., 2014). However, when the total frequencies with which the target and competitor are chosen differ across choices sets, the above procedure runs into problems. For example, assume that a participant chose Target_1_ 30 times, Competitor_1_ 20 times, Target_2_ 10 times, and Competitor_2_ 15 times (the indices correspond to Choice Sets 1 and 2, respectively) in the hypothetical car scenario presented above (cf. Table 1). Here, the RST_1_ in Context 1 is .60 and the RST_2_ in Context 2 is .40, so that the average is .5. However, following Equation 1, RST$_{\text {UW}} = \frac {30+10}{30+20+10+15} = .53$, indicating a small compromise effect. Note that in this case, the IIA was not violated since the average RST was .5.

Collapsing choice observations across the two sets (as in Eq. 1) is mathematically equivalent to calculating the RST based on the weighted average between the two within-set RST proportions, where the weights are the sample sizes of the two choice sets (see Appendix A for more details). For this reason, we call this method of measurement RST_UW_, because it allows for unequal weights.

If the total frequencies with which the target and competitor are chosen are different across the two choice contexts (i.e., if *n*_t,C1_ + *n*_c,C1_≠ *n*_t,C2_ + *n*_c,C2_), the RST should be determined as

2 $$RST_{\;\text{EW}} = 0.5*\left(\frac{n_{\mathrm{t},\mathrm{C}1}} {n_{\mathrm{t},\mathrm{C}1} + n_{\mathrm{c},\mathrm{C}1}}+ \frac{n_{\mathrm{t},\mathrm{C}2}} {n_{\mathrm{t},\mathrm{C}2} + n_{\mathrm{c},\mathrm{C}2}}\right).$$

Equation 2 is the simple average of each within-set RST (cf. Spektor et al., 2019). Because the simple average weights each sample size equally, it is denoted as RST_EW_. In our car example, RST$_{\text {EW}} = .5*(\frac {30}{30+20} + \frac {10}{10+5}) = .50$, which now correctly shows no compromise effect. RST_EW_ is therefore equally informed by the uncertainty of both within-set RST ratios, namely, RST_1_ and RST_2_. Note that when the total frequency of choosing the target and competitor is equal in both contexts, RST_EW_ and RST_UW_ are identical.

Several studies have used the RST_UW_ in recent years (e.g., Trueblood et al., 2015; Trueblood et al., 2014; Trueblood, 2012; Trueblood et al., 2013; Berkowitsch et al., 2014; Spektor, Kellen, & Hotaling, 2018; Liew et al., 2016; Evans, Holmes, Dasari, & Trueblood, 2021). Importantly, none of these studies has examined the assumption that the choice frequencies of both target and competitor are equal across different choice sets. Although a few studies have used variants of RST_EW_ (e.g., Spektor et al., 2019; Turner et al., 2018; Molloy, Galdo, Bahg, Liu, & Turner, 2019), they did so by citing Berkowitsch et al. (2014), who used the RST_UW_ measure instead. None of the aforementioned studies has questioned or examined the difference between RST_UW_ and RST_*EW*_ and their implications for statistical inference in a systematic way. In the next section, we use a simulation study to address this issue. Crucially, we propose a Bayesian formulation of the RST_EW_ for the first time.

## A simulation study of RST measures

In the previous section, we showed that the simplified RST_UW_ measure can lead to incorrect inferences about possible violations of IIA, so that RST_EW_ should always be preferred. However, RST_UW_ has been used often in past work, so it is worthwhile to question whether the approximation of RST_UW_ can lead to substantially biased inferences. We addressed this question via simulations. In the following, we show (i) how large the choice frequency differences in the two choice sets have to be so that RST_UW_ leads to biased inferences, and (ii) whether RST_UW_’s bias is affected by the strength of the underlying context effect. To do this, we simulated a population of subjects under different target/competitor sample size and effect size manipulations. We then tested whether RST_UW_ and RST_EW_ identify the true underlying context effect in the population.

We employed Bayesian and frequentist hypothesis tests. From the frequentist family, we used the *t* test (since this test has been used for RST in the literature before), which evaluates whether the mean RST of participants is equal to 50%. As a Bayesian test, we used a hierarchical version of the binomial distribution based on previous work (Trueblood, 2015). We simulated different scenarios, varying the presence or absence of the context effects and the sample-size difference between the two choice contexts.

Specifically, for the RST_UW_ version, we assumed that each participant’s RST is represented by a binomial (success) rate parameter *𝜃* and that all individual *𝜃* are sampled, at the group level, from a beta distribution with mean parameter *μ* (which also has a beta distribution) and a concentration parameter *κ* (which has a gamma distribution). We used a similar parameterization and the same prior distributions as in Trueblood (2015) and Trueblood et al. (2015). The mean and the concentration parameters were related to the alpha and beta parameters of the RST beta distribution as follows: *a* = *μκ* and *b* = (1 − *μ*)*κ*. For the RST_EW_ version, we estimated separate individual and group-level *𝜃* s across the two sets (i.e., *𝜃*_1_, and *𝜃*_2_ at the individual level, with mean and concentration *μ*_1_, *μ*_2_, *κ*_1_, and *κ*_2_ at the group level). For a graphical representation of the structure of both hierarchical models, see Appendix B.^1^

To test the context effects in the Bayesian framework, we used Bayes factors (BFs), which quantify how much more likely the data are under the alternative hypothesis than under the null (or vice versa). Crucially, and unlike *p* values, BFs can quantify evidence in favor of the null hypothesis, and not just in favor of the alternative hypothesis (Kass & Raftery, 1995; Lee & Wagenmakers, 2014; Wagenmakers et al., 2018; Aczel et al., 2018). In practice, to calculate BFs, we separately fit two models: the alternative hypothesis model, in which all previously described parameters were free to vary and were therefore estimated from the data, and the null hypothesis model, which is a constrained version of the first. In particular, in the case of RST_UW_, *μ* = 0.50 and was not estimated, and in the case of RST_EW_, *μ*_2_ = 1 − *μ*_1_, which is equivalent to setting their average to 0.50 [(*μ*_1_ + *μ*_2_)/2 = 0.50].

**Fig. 1:**
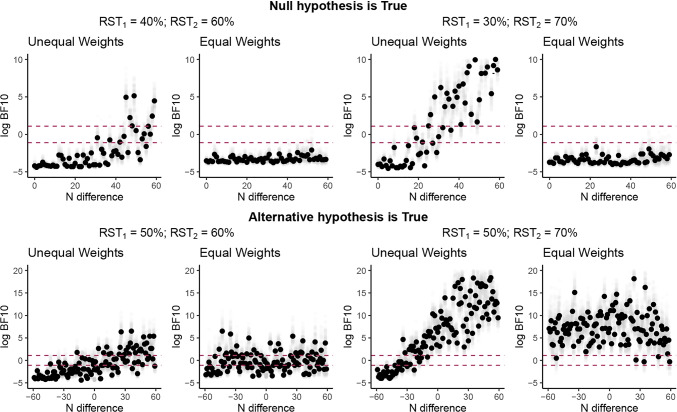
Results of simulation. The log Bayes factors (BFs) are presented in the two different RST methods (RST_UW_ and RST_EW_). *Black dots* indicate means per unit *N* difference; *gray dots* indicate raw BFs. Means are given with confidence intervals. The *dashed lines* indicate the thresholds *BF*_10_ = 3 and *BF*_10_ = 1/3. RST = Relative choice share of the target (the index indicates Set 1 or 2, respectively); EW = equal weights; UW = unequal weights. *Upper panel*: Null hypothesis is true. *Bottom panel*: Alternative hypothesis is true

We simulated different data sets, varying (1) the generating group-level binomial-rate mean parameters, and (2) the magnitude of the sample-size difference between the two choice sets. For each sample-size-difference level, we simulated 100 data sets and performed 100 independent hypothesis tests. In total, there were 59 sample-size-difference levels: For one, both sets had the same number of observations (i.e., 60 in one and 60 in the other), and for the rest we kept the sample size of one fixed (to 60 observations) and changed the sample size of the other by one unit until we had only one observation (i.e., 59, 58, ..., until 1). To use realistic generating parameter values, we took the mean posterior concentration *κ* parameter of the RST_EW_ measure applied to the data of Trueblood et al. (2015), which is a recent study on context effects (also included in our reanalysis study). We also assumed 60 observations maximum per set, which was the average participant set sample size in Trueblood et al. (2015). Specifically, we simulated 55 participants and fixed the concentration parameter of the parent beta distribution to 5. We further fixed the mean of the parent beta distribution to different RST levels such as 50, 55, and 60% to simulate different effect-size scenarios of context effects (see Fig. 1 for more details). Finally, we assumed that the sample-size difference was not the same across all participants but came from a truncated normal distribution with the intended sample-size difference as mean and a standard deviation equal to 5, to create realistic scenarios.

The simulation was performed in R. The Bayesian models were estimated using rstan through the No-U-Turn sampler (Carpenter et al., 2017). For the sampling procedure, we ran three independent chains of 1500 posterior samples each, 500 of which were used as warm-up and therefore discarded (Carpenter et al., 2017; Gelman, Carlin, Stern, Duson, & Vehtari, 2013). For the RST_UW_ measure, we adopted the same prior distributions proposed by Trueblood et al. (2015) and Trueblood (2015). The marginal likelihoods of the models were estimated through the bridge-sampling method (Gronau, Singmann, & Wagenmakers, 2020; Gronau et al., 2017). The marginal likelihoods are normalizing constants of the joint posterior distributions and are necessary to calculate BFs. They often involve calculations that lack analytical solutions. Bridge sampling can approximate BFs more accurately than other methods, such as the (naive version of the) Savage–Dickey density ratio (e.g., see Heck, 2019).

In the following, we report the results of the Bayesian analyses (for the results of the frequentist analyses, see supplementary materials). Figure 1 shows the results of the simulation where the two sets have unequal sample sizes and, therefore, where RST_EW_ and RST_UW_ diverge.

As expected, when the null hypothesis was true (i.e., when there was no violation of IIA in the generating data), RST_UW_ showed a bias in favor of the alternative hypothesis and the bias grew with higher differences in sample sizes between the two choice sets. This bias was exacerbated when the binomial rate parameter of the two sets was closer to 0 or 1. On the other hand, RST_EW_ showed no bias toward the alternative hypothesis, irrespective of sample-size differences between the two context sets. The RST_UW_ measure was also biased toward the null hypothesis when the alternative hypothesis was true (i.e., when there was a violation of IIA in the generating data), whereas the RST_EW_ measure still remained unbiased. Crucially, the RST_UW_ measure is not always biased toward the alternative hypothesis: It can, in fact, be biased either way, depending on which choice set has the largest sample size. For example, RSTs that are closer to .50 and come from the set with the larger sample size will drag the RST_UW_ toward .50, biasing the result toward the null hypothesis. Alternatively, RSTs of one set that are not closer to .50 and have a larger sample size than the other set will push RST_UW_ away from .50, thus biasing the results toward the alternative hypothesis. The larger the sample-size differences across sets, the more biased the results of the RST_UW_ measure were. In sum, our simulations showed that the RST_UW_ measure produces false negatives or false positives when the total number of target and competitor choices is unequal across the two choice contexts.^2^

## Reanalyses of past studies

The simulations showed that RST_UW_ is a biased measure of context effects whereas RST_EW_ proves to be a robust measure. To examine whether RST_UW_ might have led to inaccurate conclusions in previous studies, we reanalyzed the data of five published articles using both RST_UW_ and RST_EW_ measures and evaluated their agreement.

To select which studies to reanalyze, we searched for original research articles examining the attraction, similarity, and compromise effects among all issues of four major psychological journals (*Journal of Experimental Psychology: General*, *Psychological Review*, *Psychological Science*, *Psychonomic Bulletin & Review*) published in the past decade (2010–2019). First, we identified 811 articles that contained the following keywords in their title and/or abstract: context (effect), attraction (effect), compromise (effect), similarity (effect). From this set of articles, we selected only those 17 in which options were characterized by more than one attribute. From this list, we selected five (i.e., Berkowitsch et al., 2014; Liew et al., 2016, Trueblood et al. 2015, 2014, Cataldo & Cohen, 2019) that had original data examining all three effects using a within-subject design (see supplementary materials for details). The within-subject design was necessary to examine the presence of correlations between context effects because of their hypothesized theoretical importance (e.g., Berkowitsch et al., 2014; Trueblood et al., 2015).

The study of Berkowitsch et al. (2014) and Study 2 from Liew et al. (2016) involved preferential tasks where individuals could choose between consumer products (e.g., notebook computers) with different attributes (e.g., weight in kilograms and battery life in hours). Although the study of Liew et al. (2016) is a replication of that of Berkowitsch et al. (2014), the former has a considerably larger pool of subjects (i.e., 134, compared to 48 in the original study). Cataldo and Cohen (2019) tested the effect of the presentation format on context effects in preferential tasks as well. We focus here on their results from the condition of by-alternative (vs. by-attribute) presentation format of stimuli, because this is more comparable with the rest of the studies included in our reanalysis. The study of Trueblood et al. (2015), on the other hand, involved a perceptual decision-making task where participants had to correctly indicate which rectangle had the largest area, given their different length and height attributes. Finally, in the study of Trueblood et al. (2014), participants were asked to indicate the likely murderer from a triplet of suspects.

The five studies included for reanalysis are of empirical importance because two of them (Trueblood et al., 2015, 2014) have been the basis for development of cognitive modeling, and one (Berkowitsch et al., 2014) rigorously tested different psychological models. In addition, all studies represent context-effect research in different domains: perceptual discrimination, suspect judgment, and consumer decisions. Finally, four of them (i.e., Berkowitsch et al., 2014, Trueblood et al. 2015, 2014; Liew et al., 2016) used the RST_UW_ as a measure of context effects.

To preprocess the data of the five studies, we applied the procedures that were described in the relative published articles. Thus, we performed our analyses on the same data sets that were originally analyzed. Our reanalyses consisted of the same two measures that we utilized in the simulations: RST_UW_ and RST_EW_. Specifically, we estimated the BF in favor of the null (i.e., no IIA violation) and alternative (i.e., IIA violation) hypotheses, separately for both RST measures. We estimated all Bayesian models separately for context effect and study. Overall, we thus fitted 60 Bayesian models (3 context effects × 5 studies × 2 RST measures × 2 hypotheses). All parameters of the Bayesian models were estimated in Stan (Carpenter et al., 2017) through the No-U-Turn sampler with six independent chains, each of which consisted of 20,000 posterior samples where the first 1000 were discarded as warm-up (all other settings of model structure and fitting were the same as in our simulation study; for details see Appendix B). The prior distributions of the Bayesian models were the same as in Trueblood et al. (2015). All models converged with $\hat {R}$ always lower than 1.01.

In our simulations, the two RST measures led to the same inferences when there was no sample-size difference across the two core option sets, but they diverged when these differences were substantial. However, by reanalyzing previously published data, we aimed at understanding how large these differences are in empirical data and whether such differences can also lead to biased RST conclusions. Substantial differences might especially occur in the similarity and compromise effect conditions. This is because the third added option could represent an attractive option (as it is more extreme on one attribute’s scale) and the attractiveness of the third option could vary across sets because of different attribute preferences. This may lead to different sample sizes of the core options. In contrast, in the attraction effect, the dominated decoy option is not chosen very often, leading to similar sample sizes for the two core options. Therefore, we predicted that the two RST measures would not substantially diverge in the attraction effect condition but would disagree in the similarity and compromise effect conditions.

**Fig. 2:**
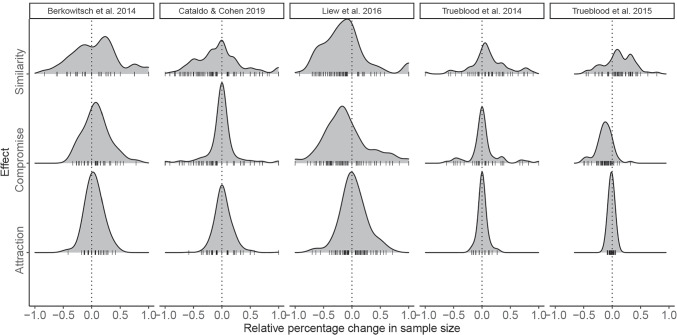
Empirical density distributions of relative percentage change in the sample size across the two choice sets. *Ticks* below the distributions indicate raw observations. Zero is marked by a *dotted line*

We examined the sample-size differences between the two sets across all studies and context effects (Fig. 2). Densities of sample-size differences that are centered at zero indicated no substantial sample-size differences. Non-zero-centered distributions indicated that one set has more observations than the other on average. As we expected, the distributions of the attraction effect were mostly centered around zero. On the other hand, the density distributions in the similarity and compromise effects in the Liew et al. (2016), and Trueblood et al. (2015, 2014) studies were shifted away from zero.

### Results from highest density intervals

To perform hypothesis testing, we looked at the 95% highest density intervals (HDI_95*%*_) of the posterior distributions of the hierarchical RST mean. Unlike for the simulation study, where the goal was to evaluate the strength of evidence in favor of or against the null/alternative hypotheses under RST_EW_ and RST_UW_, we focus on the HDIs of the RST measure in this section, since HDIs provide information about the effect size. Specifically, HDIs not only clarify if there is an effect but they also provide information about the strength and direction of the effect (i.e., if RST is below or above 50%). Therefore, we report the results of hypothesis testing of HDIs in the main text and, for simplicity, BFs in Appendix C (we followed the same procedures to derive BFs as in our simulation study; cf. Lee & Wagenmakers, 2014; Kass & Raftery, 1995; Gelman et al., 2013; Kruschke & Liddell, 2018; Wagenmakers et al., 2018; Dienes, 2016).

**Fig. 3:**
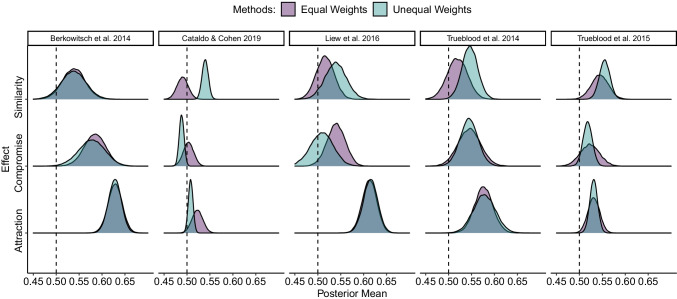
Posterior distributions of the hierarchical mean RST in the five studies included for reanalysis. The posteriors of both the RST_UW_ and the RST_EW_ measure are presented. In the RST_UW_ measure, the hierarchical mean is plotted directly, whereas in the RST_EW_ measure, the hierarchical mean is computed as the arithmetic average of the mean hierarchical posteriors of the two sets. RST = Relative choice share of the target; EW = equal weights; UW = unequal weights

Figure 3 shows the posterior distributions of the group-level mean of the RST measures. Table 2 provides their respective HDIs. For the attraction effect, both RST_UW_ and RST_EW_ led to the same qualitative conclusions in all five studies. Four of the five studies supported the presence of an attraction effect (i.e., the mean posterior of both RST measures did not include 50%); the study of Cataldo and Cohen (2019) did not.

**Fig. 4:**
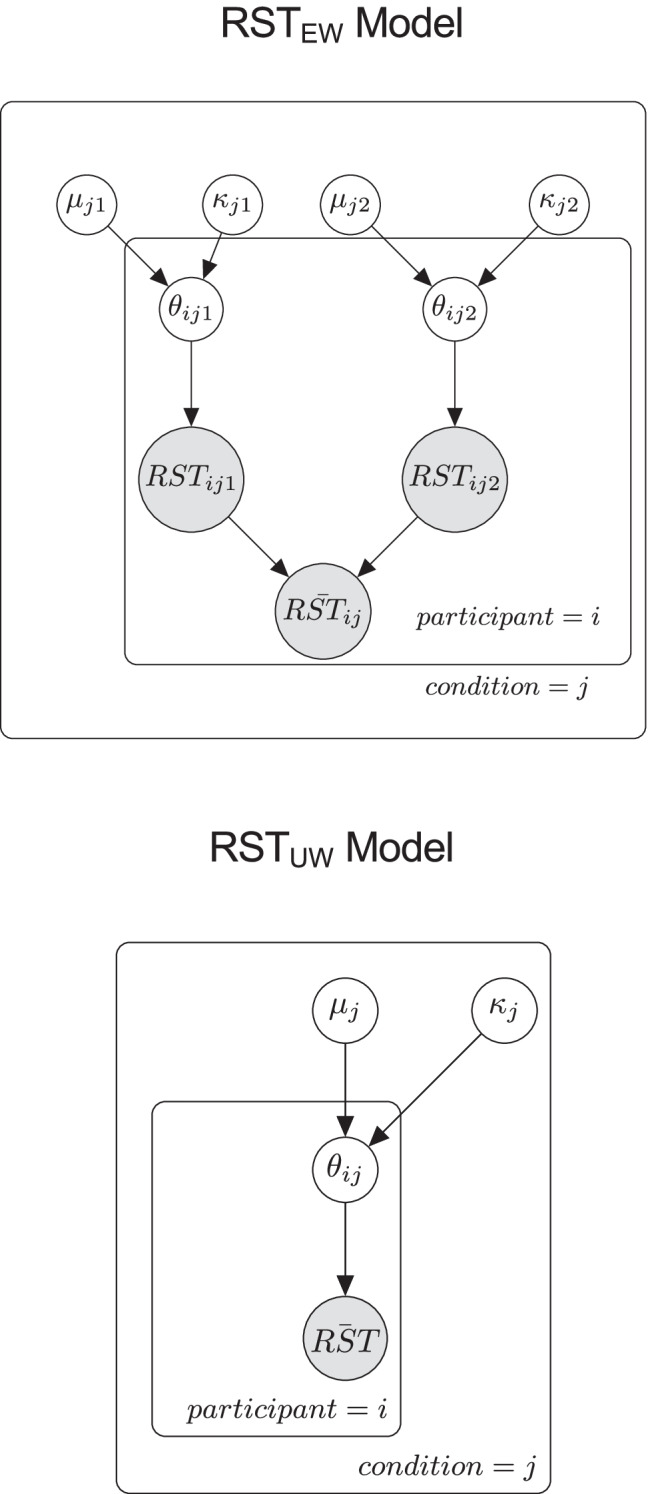
Structure of the hierarchical relative choice share of the target (RST) measures. *Upper panel*: RST_EW_ measure of the alternative hypothesis, where *μ* and *κ* indicate the hierarchical mean and concentration parameters, the index (1 or 2) indicates the set, and *𝜃* reflects each participant’s RST binomial rate parameter within each set (1 and 2). $\bar {RST}$ is the arithmetic average of the two within-set RSTs. In the null hypothesis, *μ*_*j*2_ is constrained as *μ*_*j*2_ = 1 − *μ*_*j*1_, and it is not freely estimated. *Lower panel*: RST_UW_S measure of the alternative hypothesis, where *μ* and *κ* indicate the hierarchical mean and concentration parameters. The measure does not demarcate which observations come from which choice set, because it collapses the observations across sets into one distribution. Again, *𝜃* reflects each participant’s RST binomial parameter. In the null hypothesis, *μ*_*j*_ is set to 0.50 and it is not estimated. EW = Equal weights; UW = unequal weights

As expected, a disagreement between the two RST measures was observed for the compromise and similarity effects: In the study by Trueblood et al. (2014), a compromise effect was identified when relying on RST_UW_ but not when using the unbiased RST_EW_ measure, whereas in Liew et al. (2016), RST_EW_ established the compromise effect in contrast to RST_UW_. In the other three studies, identical conclusions regarding the compromise effect were drawn when relying on either measure. Concerning the similarity effect, in the studies by Cataldo and Cohen (2019) and Trueblood et al. (2014), a similarity effect was identified when relying on RST_UW_ but not when using the accurate RST_EW_ measure. In contrast, in the other three studies, identical conclusions regarding the similarity effect were drawn when relying on either measure Fig. 4.

In sum, the RST_UW_ measure can lead to incorrect inferences. Overall, we tested three effects in five studies across a variety of decision domains. Both measures led to the same qualitative results in 11 of 15 cases of context effects. However, in one-fourth of the studies (i.e., four cases) where RST_UW_ erroneously established an effect were the similarity effect in Cataldo and Cohen (2019) and Trueblood et al. (2014), and the compromise effect in Liew et al. (2016) and Trueblood et al. (2014). Generally, the comparison based on the HDIs reveals that the RST_EW_ measure tends to establish posterior RST means with higher uncertainty. Moreover, the RST_EW_ measure led to mean RST posterior distributions that included 50% more often compared to RST_UW_, in the similarity and compromise effect conditions. In only a few cases did the RST_EW_ measure conclude that the mean RST posterior is not 50%, in contrast to the conclusions of the RST_UW_ measure.

**Table 2:** Upper and lower 95% HDI cutoffs of posterior distributions of the hierarchical mean RST distributions by study, method (unequal vs. equal weights), and context effect

Study	Attraction	Compromise	Similarity
Unequal weights			
Berkowitsch et al. (2014)	[0.6, 0.655]	[0.52, 0.634]	[0.485, 0.591]
Cataldo and Cohen (2019)	[0.498, 0.518]	[0.478, 0.498]	[0.526, 0.553]
Liew et al. (2016)	[0.584, 0.646]	[0.457, 0.556]	[0.493, 0.584]
Trueblood et al. (2014)	[0.532, 0.626]	[0.505, 0.583]	[0.512, 0.58]
Trueblood et al. (2015)	[0.515, 0.548]	[0.498, 0.539]	[0.532, 0.577]
Equal weights			
Berkowitsch et al. (2014)	[0.599, 0.659]	[0.538, 0.631]	[0.49, 0.587]
Cataldo and Cohen (2019)	[0.499, 0.546]	[0.48, 0.525]	[0.466, 0.514]
Liew et al. (2016)	[0.582, 0.644]	[0.501, 0.579]	[0.477, 0.554]
Trueblood et al. (2014)	[0.538, 0.617]	[0.497, 0.595]	[0.475, 0.562]
Trueblood et al. (2015)	[0.506, 0.556]	[0.481, 0.564]	[0.508, 0.582]

Values are rounded to the third decimal. HDI = highest density interval; RST = relative choice share of the target

So far we have examined the extent to which RST_EW_ and RST_UW_ make the same inferences when applied to the data of the five articles that were included in the reanalysis following our Bayesian approach. However, if one used the accurate RST_EW_ measure, the qualitative results of the statistical inference might coincide with the conclusions reported in the original papers. For this reason, we compared the inferential results of the RST_EW_ measure to the originally reported statistics (cf. Table 3).

To make the comparison more direct, we followed the same statistical test and framework (i.e., frequentist or Bayesian) of the original studies while switching from RST_UW_ to RST_EW_. Specifically, for Trueblood et al. (2014) we used a one-sample frequentist *t* test on RST_EW_, for Liew et al. (2016) we used a Bayesian one-sample *t* test on RST_EW_, and for Berkowitsch et al. (2014) and Trueblood et al. (2015) we used our proposed RST_EW_ Bayesian measure (because the last two studies used a Bayesian formulation of the RST_UW_). In all Bayesian analyses, we employed the same prior distributions reported in the original articles. Finally, we did not include Cataldo and Cohen (2019) in the comparison for the following reason: The authors used a regression model with several main effect and interaction terms. Because we looked only at a subset of their data (i.e., by-alternative presentation condition), and given that regression coefficients are conditional on the data and the regression terms included in the model, we excluded the study from the comparison.

**Table 3:** Comparison of RST_EW_ reanalysis results and originally reported test results

Study	Attraction	Compromise	Similarity	Framework
Reanalysis results				
Berkowitsch et al. (2014)	*HDI*_95*%*_ = [0.598, 0.659]	*HDI*_95*%*_ = [0.539, 0.632]	*HDI*_95*%*_ = [0.489, 0.586]	Bayesian RST_EW_
Liew et al. (2016)	$HDI_{95\%}^{\delta }$ = [0.413, 0.823]	$HDI_{95\%}^{\delta }$ = [-0.024, 0.360]	$HDI_{95\%}^{\delta }$ = [-0.148, 0.219]	Bayesian *t* test
Trueblood et al. (2014)	*t*(64) = 3.20, *p* = .002	*t*(63) = 2.40, *p* = .019	*t*(63) = 0.79, *p* = .43	frequentist *t* test
Trueblood et al. (2015)	*HDI*_95*%*_ = [0.506, 0.556]	*HDI*_95*%*_ = [0.481, 0.564]	*HDI*_95*%*_ = [0.508, 0.582]	Bayesian RST_EW_
Reported test result				
Berkowitsch et al. (2014)	*HDI*_95*%*_ = [0.60, 0.66]	*HDI*_95*%*_ = [0.52, 0.63]	*HDI*_95*%*_ = [0.48, 0.59]	Bayesian RST_UW_
Liew et al. (2016)	$HDI_{95\%}^{\delta }$ = [0.45, 0.87]	$HDI_{95\%}^{\delta }$ = [0.03, 0.40]	$HDI_{95\%}^{\delta }$ = [-0.13, 0.23]	Bayesian *t* test
Trueblood et al. (2014)	*t*(64) = 3.14, *p* = .003	*t*(64) = 2.17, *p* = .034	*t*(64) = 2.58, *p* = .012	frequentist *t* test
Trueblood et al. (2015)	*HDI*_95*%*_ = [0.514, 0.548]	*HDI*_95*%*_ = [0.498, 0.538]	*HDI*_95*%*_ = [0.532, 0.578]	Bayesian RST_UW_

RST = relative choice share of the target; EW = equal weights; UW = unequal weights; *HDI*_95*%*_ = 95% highest density interval of the posterior hierarchical mean RST distributions according to RST_EW_ or RST_UW_ used in the simulation study; $HDI_{95\%}^{\delta }$ = highest density interval of the effect size of a one-sample Bayesian *t* test. Values are rounded to the third decimal

Table 3 shows that in all the studies in which the RST_UW_ was used (i.e., Berkowitsch et al., 2014; Trueblood et al., 2015; 2014; Liew et al., 2016), no evidence for the alternative hypothesis (i.e., that RST is not equal to 50%) was found under the RST_EW_ measure in five of the total of 12 cases. Specifically, the attraction effect was supported in all studies (i.e., Berkowitsch et al., 2014; Trueblood et al., 2015; 2014; Liew et al., 2016), whereas the compromise effect was observed only in Berkowitsch et al. (2014) and Trueblood et al. (2014).^3^ On the other hand, the similarity effect was found only in the study of Trueblood et al. (2015). Generally, the unbiased RST_EW_ measure made different qualitative conclusions than those originally reported in two of 12 cases (i.e., in the compromise effect of Liew et al., 2016, and in the similarity effect of Trueblood et al., 2014).

### Correlations among context effects

To understand the underlying cognitive mechanisms driving the effects, research on context effects has also examined the correlation between effects. In previous studies, a positive correlation between the attraction and compromise effects has been observed, along with negative correlations between the compromise and similarity effects, and between the similarity and attraction effects (e.g., Berkowitsch et al., 2014). These specific correlations play a significant role in the evaluation of psychological theories, because their existence might imply that similar cognitive mechanisms cause certain context effects (for a recent large-scale replication of these correlations, see Dumbalska, Li, Tsetsos, & Summerfield, 2020). For this reason, we determined the correlations among effects for the five studies we reanalyzed (see Table 4 for HDIs of correlation coefficients). We found that the correlation coefficients were mostly similar between the RST_UW_ and RST_EW_ measures with the only exception being the correlation between the similarity and compromise effects in Trueblood et al. (2015).

Therefore, in contrast to the RST analysis presented above regarding the population mean of the RST, the two RST measures largely agreed in their qualitative conclusions regarding the correlation between the context effects. This happened for two reasons. First, correlations of two variables are modeled according to the multivariate normal distribution (which has marginal means and the variance–covariance matrix as parameters; marginal refers to the parameter or distribution of one of the two variables that enter the correlation). Correlations are not affected by changes in the location of the marginal means (e.g., the mean of the two marginal distributions whose correlation we estimate). Therefore, although RST_EW_ and RST_UW_ might disagree on the RST mean of the group, such disagreement might not affect the correlation coefficients.

Second, correlations can be affected by differences in the marginal variance of RST distributions (i.e., larger variance differences across marginal distributions render correlations more and more difficult to find). We evaluated the marginal variance of the group-level RST distributions for both RST_EW_ and RST_UW_ (for more details see the supplementary materials) and we found that the two measures produced similar marginal variances for each context effect, thus preserving the effect covariation. This explains why the two RST measures produced similar qualitative results regarding correlations. We can, therefore, conclude that context-effect correlations are generally a more robust pattern than RSTs, even in the presence of sample-size differences across choice sets.

**Table 4:** Coefficients for context-effect correlations by study and RST method

Study	Com vs. Att	Com vs. Sim	Sim vs. Att
Unequal Weights			
Berkowitsch et al. (2014)	*r* = .43; [.216, .641]	*r* = -.52; [-.712, -.315]	*r* = -.48; [-.681, -.264]
Cataldo and Cohen (2019)	*r* = .29; [.204, .377]	*r* = -.39; [-.478, -.315]	*r* = -.33; [-.408, -.242]
Liew et al. (2016)	*r* = .57; [.446, .693]	*r* = -.76; [-.841, -.686]	*r* = -.53; [-.654, -.396]
Trueblood et al. (2014)	*r* = .29; [.076, .504]	*r* = -.22; [-.434, .005]	*r* = -.38; [-.566, -.17]
Trueblood et al. (2015)	*r* = .63; [.456, .774]	*r* = -.25; [-.487, -.016]	*r* = -.26; [-.495, -.035]
Equal weights			
Berkowitsch et al. (2014)	*r* = .44; [.216, .649]	*r* = -.49; [-.687, -.287]	*r* = -.45; [-.662, -.231]
Cataldo and Cohen (2019)	*r* = .23; [.139, .319]	*r* = -.22; [-.314, -.136]	*r* = -.26; [-.346, -.17]
Liew et al. (2016)	*r* = .55; [.41, .671]	*r* = -.77; [-.843, -.694]	*r* = -.55; [-.676, -.419]
Trueblood et al. (2014)	*r* = .39; [.189, .572]	*r* = -.12; [-.352, .106]	*r* = -.32; [-.536, -.116]
Trueblood et al. (2015)	*r* = .47; [.273, .659]	*r* = -.16; [-.389, .099]	*r* = -.3; [-.519, -.076]

The mean posterior *rho* is indicated by *r*. The 95% highest density intervals are also given. Att = attraction effect; Sim = similarity effect; Com = compromise effect; RST = relative choice share of the target. Values are rounded to the third decimal

## A note on detecting violations of the regularity principle

So far, we have discussed two ways of identifying violations of the IIA principle in a sample, namely RST_EW_ and RST_UW_. We showed that RST_EW_ has advantages over RST_UW_ and that published studies that used the RST_UW_ can, sometimes, lead to erroneous inferences.

The *regularity* principle (Luce, 1977) is conceptually related to (but not logically implied by) the IIA. According to regularity, adding an option to a choice set should never increase the choice probabilities of the options from the original set (for a review see Rieskamp et al., 2006). The attraction effect was historically taken as an instance of an empirical illustration of a violation of the regularity principle (Huber et al., 1982). In contrast with the proposed RST_EW_ measure—which tests only for violations of the IIA principle—we introduce a measure appropriate for testing violations of the regularity principle. This measure should be used specifically to test the presence of the attraction effect.

### The absolute choice share of the target and competitor

Formally, the regularity principle states that for any option *x* that is part of the option sets *X* and *Y* it should hold that when $X \subseteq Y$, *P*_*X*_(*x*) ≥ *P*_*Y*_(*x*). A direct test for the regularity principle is to use a one-pair-one-triplet experimental design, where participants initially express preferences for two options and then again after a third option is added to the choice set. In this design, a direct violation of the regularity principle occurs if the probability of either option originating from the pair set increases in the triplet set. Many studies have used this design, including the original attraction effect study (i.e., Huber et al., 1982).

However, the focus of the present work is the two-triplet design. In this design, two options *A* and *B* are embedded in two different triplets. Each triplet is made with the addition of a decoy option: *D*_1_, which is close to the target option *A* (Context 1; C1) and *D*_2_, which is close to the target option *B* (Context 2; C2). Although in this design the choice probabilities of *A* and *B* in the pair {*A*,*B*} are never observed, we can deduce relations between the two-triplet choice sets if regularity holds (as shown in Appendix D in more detail). Therefore, the two-triplet design can indirectly test for violations of the regularity principle.

Specifically, we propose the *absolute choice share of the target* (AST) and *absolute choice share of the competitor* (ASC) as measures for the attraction effect and the reversed attraction effect (for details about their derivation see Appendix D):

3 $$AST = 0.5*\left(\frac{n_{\mathrm{t},\mathrm{C}1}} {n_{\mathrm{t},\mathrm{C}1} + n_{\mathrm{c},\mathrm{C}1} + n_{\mathrm{d},\mathrm{C}1}}+ \frac{n_{\mathrm{t},\mathrm{C}2}} {n_{\mathrm{t},\mathrm{C}2} + n_{\mathrm{c},\mathrm{C}2}+ n_{\mathrm{d},\mathrm{C}2}}\right),$$

4 $$ASC = 0.5*\left(\frac{n_{\mathrm{c},\mathrm{C}1}} {n_{\mathrm{t},\mathrm{C}1} + n_{\mathrm{c},\mathrm{C}1} + n_{\mathrm{d},\mathrm{C}1}}+ \frac{n_{\mathrm{c},\mathrm{C}2}} {n_{\mathrm{t},\mathrm{C}2} + n_{\mathrm{c},\mathrm{C}2}+ n_{\mathrm{d},\mathrm{C}2}}\right),$$

where *n*_t_, *n*_c_ and *n*_d_ refer to the choice frequencies of the target (t), competitor (c), and decoy (d), respectively. Regularity is satisfied if both AST and ASC are below or equal to 50%. AST > 50% indicates the presence of an attraction effect, whereas ASC > 50% indicates the presence of the reverse of the attraction effect.^4^ Note that AST ≤ 50% or ASC ≤ 50% alone does not necessarily imply no regularity violation. Therefore, if one is agnostic about the hypothesized direction of the regularity violation, one should look at both AST and ASC to see if either of them is above 50%.

### Reanalyses of past studies

Although the RST is different from the AST (as the former evaluates violations of the IIA principles in the similarity and compromise effects, and the latter evaluates violations of the regularity principle in the attraction effect), many studies that employed two-triplet experimental designs have instead used the RST to analyze attraction effect trials (e.g., Spektor et al., 2018; Trueblood et al., 2013; Berkowitsch et al., 2014; Trueblood et al., 2015; Trueblood, 2012; Spektor et al., 2019; Evans et al., 2021, among others). In this section, we propose a Bayesian formulation of AST and ASC and, furthermore, we apply the AST to the data of published studies.

We created a Bayesian model to infer AST and ASC from a sample, which is an extension of the Bayesian formulation of the RST_EW_. Specifically, we modeled each participant’s choice probabilities as a multinomial simplex vector $\vec {\theta }$. All participants’ $\vec {\theta }$ were constrained from a group-level Dirichlet distribution with a simplex mean vector $\vec {\mu }$ and a concentration parameter *κ*. $\vec {\mu }$ and *κ* followed the Dirichlet and the gamma distribution, respectively. We used a Dirichlet prior of (2,2,2) on $\vec {\mu }$, which is the multinomial-equivalent of the prior we used for the RST_EW_ measure in case of three alternatives. For *κ*, we employed a gamma prior of (0.001,0.001), which is the same that we used for the RST_EW_ measure as well. Crucially, as with the RST_EW_ measure, we estimated different hierarchical and low-level parameters across the two choice sets. Therefore, AST is derived from the average between the posterior of the target in $\vec {\mu _{1}}$ (i.e., from Set 1) and the posterior of the target in $\vec {\mu _{2}}$ (i.e., from Set 2), and similarly for ASC but with the posteriors of the competitor option.

Table 5 presents the results of AST reanalysis of the studies that were used in the reanalysis of RST_EW_ in the previous section (i.e., Berkowitsch et al., 2014; Trueblood et al., 2015; 2014; Liew et al., 2016). In one of four cases (i.e., Trueblood et al., 2015), no evidence of the attraction effect was found under AST, whereas the alternative hypothesis (i.e., that AST is higher than 50%) of an attraction effect was supported in the original study. For all other studies, the qualitative results of the AST measure corresponded to that originally reported. Generally, under AST, the strength of the attraction effect was less strong (i.e., the mean posterior distributions were closer to the null hypothesis).

**Table 5:** Comparison of AST reanalysis results in attraction effect trials and originally reported test results

Study	Reanalysis result	Reported test result
Berkowitsch et al. (2014)	*HDI*_95*%*_ = [0.578, 0.640]	*HDI*_95*%*_ = [0.60, 0.66]
Liew et al. (2016)	$HDI_{95\%}^{\delta }$ = [0.060, 0.447]	$HDI_{95\%}^{\delta }$ = [0.45, 0.87]
Trueblood et al. (2014)	*t*(64) = 2.25, *p* = 0.013	*t*(64) = 3.14, *p* = 0.003
Trueblood et al. (2015)	*HDI*_95*%*_ = [0.482, 0.526]	*HDI*_95*%*_ = [0.514, 0.548]

AST = absolute choice share of the target; *HDI*_95*%*_ = 95% highest density interval of the posterior hierarchical mean relative choice share of the target (RST) distributions according to the RST_EW_ (EW = equal weights); $HDI_{95\%}^{\delta }$ = highest density interval of the effect size of a one-sample Bayesian *t* test. Both the Bayesian and the frequentist *t* tests were one-sided. Values are rounded to the third decimal

## Discussion

The current work examines the statistical analysis of context effects in multiattribute decision making. In particular, when determining the effect of a context in triplet designs, it is important to be aware of biases caused by differences in the choice frequency of the target and competitor options across choice sets. First, the often-used RST method for context effects (i.e., RST_UW_) is not robust to such biases as compared to an RST that calculates the pooled mean across different choice sets (i.e., RST_EW_), and second, it is not appropriate for the attraction effect, where the AST should be used instead. Furthermore, the conclusions of previously published studies changed in one-fourth of the cases when reanalyzed with robust and appropriate methods. Our results emphasize the importance of devising and evaluating statistical tests before empirically testing axiomatic principles of decision making.

Specifically, we first showed through a simulation study that different conclusions can be drawn whenever the choice frequencies for the two core options differ substantially between contexts. When the within-set RST is closer to 0 or 1, even a difference of half the sample size between the two choice sets can make the RST_UW_ approximation be biased. With within-set RST closer to .50, larger sample-size differences are required to bias RST_UW_. Second, we examined if the use of the accurate RST_EW_ would change the conclusions of past studies that had used RST_UW_. For this, we reanalyzed the data of five published studies on context effects. The results showed substantial differences: The two RST methods disagreed in 25% of the cases when considering the HDIs. In cases of disagreement, RST_EW_ mostly (but not always) favored the null hypothesis, whereas RST_UW_ indicated an effect where in fact no effect occurred. The disagreement concerned the similarity and compromise effects, where the choice frequencies can differ substantially across contexts. In cases of the similarity and compromise effect, the third options can represent an attractive option, so it might be chosen with high frequency. This can lead to large sample-size differences across contexts (cf. Fig. 2). In contrast, in cases of the attraction effect, the third option is a dominated option, so that it is rarely chosen and thus does not modify the overall choice frequencies of the two core options as much as is the case for the similarity and compromise effects.

We further looked at the differences of BFs between RST_EW_ and RST_UW_ when applied to the reanalysis of past studies (see Appendix C for more details). The BFs showed that the RST_EW_ and RST_UW_ measures disagreed in 40% of the cases, which indicates an increased disagreement rate compared to the HDIs of the two RST measures. Generally, we found that BFs were more conservative than HDIs in supporting the alternative hypothesis (cf. Wagenmakers, Lee, Rouder, & Morey, 2019).

Interestingly, when relying on the RST_EW_ measure, we observed less evidence for context effects. According to the BF analysis, at least moderate evidence for the existence of context effects was observed in only 26% of the cases, and likewise the HDIs indicate an effect in only 46% of the cases. Therefore, our results corroborate the finding that context effects can be hard to find on the aggregate level (sometimes called “the fragile nature” of context effects according to Trueblood et al., 2015). In sum, our results show that it is important to use the accurate RST_EW_ measure to identify context effects, because the RST_UW_ measure is prone to biased conclusions.

The question of how to collapse choices across different sets of options to compute the RST is also relevant in experimental designs where a baseline condition with the two core options is added to the condition with two triplet sets. For example, Turner et al. (2018) used a modified version of the RST_EW_ that adjusted for the baseline probabilities of the two core options. In addition, experimental designs that employ only a binary and a ternary choice set may avoid the question of collapsing observations since there is no target option in the binary set. Future research should thus examine and compare existing methods of hypothesis testing in these different experimental designs.

Crucially, we also make the novel contribution of illustrating that the RST measures are not suitable for identifying violations of the regularity principles. Instead, in the case of the attraction effect, the AST and ASC should be used instead of RST measures. Unlike the RST, the AST and ASC measures represent proper tests of the regularity principle. In contrast, the RST measures only test for violations of the IIA principle (i.e., similarity and compromise effects). For the purpose of hypothesis testing, we proposed a Bayesian formulation of the AST, which is a generalization of the Bayesian model of the RST_EW_ measure. In addition, after reanalyzing past studies, we observed that the attraction effect was estimated to be smaller using the AST compared to the RST measure. In one case the effect also disappeared (i.e., Trueblood et al., 2015). These results highlight the importance of employing the unbiased RST_EW_ in case of IIA violations and the AST/ASC in case of regularity violations.

Throughout our analyses we used the Bayesian framework for hypothesis testing. We did so because we believe this framework has advantages over traditional null-hypothesis significance testing (cf. Wagenmakers et al., 2018; Lee & Wagenmakers, 2014). However, the bias of the RST_UW_ measure persists even if one resorts to frequentist statistics, as we showed in our simulation (see supplementary materials). Therefore, our results are informative also for researchers who wish to implement their analyses in the frequentist framework instead.

The measures we proposed apply not only to the three popular context effects (i.e., attraction, similarity, and compromise effects) but also to additional context effects that are elicited through two ternary choice sets.^5^ As a proof of concept, we reanalyzed the data of Spektor et al. (2018), who investigated the emergence of the reversal of the attraction effect (i.e., the so-called repulsion effect) with different incentivization schemes with perceptual stimuli (for details and results see supplementary materials). Interestingly, the authors found a repulsion effect in both the gain and the loss domain of their Experiment 1. Although Spektor et al. (2018) used the RST_UW_ measure, the proper tests for violations of the regularity principle are the AST and the ASC. Our reanalysis with these absolute measures indicated that, unlike the authors’ conclusions for their Experiment 1, there was no repulsion effect in either the loss or the gain domain.

In our simulation study, we showed that RST_EW_ circumvents the problem of unequal attribute preference in context-effect experiments by modeling the RST of each choice set separately. However, we employed the RST_EW_ only as a measurement tool and not as a cognitive explanation of how attribute preferences arise (in contrast to cognitive process models such as Trueblood et al., 2014; Roe et al., 2001; Bhatia, 2013; Usher & McClelland, 2004; Noguchi & Stewart, 2018; Howes et al., 2016; Spektor et al., 2019). Researchers who are interested in explaining the cognitive underpinnings of human behavior could use the RST_EW_ measure as a starting point to empirically establish the presence of context effects before building more complex (cognitive) models to better understand the behavior of participants. Therefore, our work is of great importance for theory advancement.

Our work is in line with recent calls to revisit the assumptions of traditional statistical methods to achieve higher levels of reproducibility and statistical clarity in the field of psychology (e.g., Wagenmakers, 2007; Ioannidis, 2005; Wagenmakers, Wetzels, Borsboom, van der Maas, & Kievit, 2012; Munafó et al., 2017; Cumming, 2014; Gigerenzer & Marewski, 2015; Nuzzo, 2014). Although the field of decision making has recently seen a steep increase in cognitive models, much less attention has been paid to the methodological challenges characterizing the statistical analysis of the effects the models aim to explain. As shown in the present work, these challenges are nontrivial since they may lead to biased conclusions if they are not adequately dealt with. We believe that developing robust statistical tests that are able to conclude the presence or absence of psychological effects should be given high priority.

### Supplementary Information

ESM 1 (PDF 13423˙2022˙2157˙MOESM1˙ESM.pdf)

## Data Availability

Data and code can be found on OSF: https://osf.io/d28yz/.

## Appendix A: Relationship between the weighted average method and the sum of binomials

In this section, we show that collapsing the observations of the two sets to compute the RST_UW_ measure is equivalent to taking the weighted average between the two within-set RSTs where the weights are the sample size of each RST set.

Assume two normally distributed random variables *X* and *Y* with respective sample means $\bar {x}$ and $\bar {y}$ and sample sizes *n*_*X*_ and *n*_*Y*_. By definition, the general form of the weighted average between $\bar {x}$ and $\bar {y}$ is

A.1 $$WA = \frac{n_{X}*\bar{x} + n_{Y}*\bar{y}}{n_{X} + n_{Y}}.$$

In the case of RST, *X* and *Y* are two random binomial variables with $\hat {p}_{X}$ and $\hat {p}_{Y}$ being the unbiased maximum likelihood estimates of their respective rate parameters (*p*), and *n*_*X*_ and *n*_*Y*_ being their respective sample sizes. By definition, the weighted average of the two set proportions is

A.2 $$RST_{WA} = \frac{n_{X}*\hat{p}_{X} + n_{Y}\;\hat{p}_{Y}}{n_{X} + n_{Y}}.$$

Both $\hat {p}_{X}$ and $\hat {p}_{Y}$ can be rewritten as $\frac {s_{X}}{n_{X}}$ and $\frac {s_{Y}}{n_{Y}}$, where *s*_*X*_ and *s*_*Y*_ are the number of sample successes of each binomial variable. Therefore, Equation A2 can be reexpressed as follows:

A.3 $$RST_{WA} = \frac{n_{X}*\frac{s_{X}}{n_{X}}+ n_{Y}*\frac{s_{Y}}{n_{Y}}}{n_{X} + n_{Y}},$$

which, through simple arithmetic, can be rewritten as follows:

A.4 $$RST_{WA} = \frac{s_{X}+ s_{Y}}{n_{X} + n_{Y}},$$

which is equivalent to collapsing the observations of the two variables. QED.

## Appendix C: Bayes factor results of the reanalyses of past studies

The BF analyses followed the same procedures as the simulation analyses. A BF of 1 indicates that the null (i.e., absence of IIA violation) and alternative (i.e., presence of IIA violation) hypotheses are equally likely, BFs between 1/3 and 3 provide anecdotal evidence for either of the two hypotheses, and BFs > 3 (or BFs < 1/3) provide moderate to strong evidence for one of the two hypotheses (cf. Lee & Wagenmakers, 2014). Thus, in the following, we interpret only BFs above 3 or below 1/3 as providing evidence for one of the two hypotheses.

Table 6 shows the BF results. When examining the similarity effect, we find that the two different RST measures led to inconsistent conclusions when relying on a BF test. In Cataldo and Cohen (2019), the use of the RST_UW_ measure led to a BF that indicated a strong similarity effect, but when relying on the accurate RST_EW_ measure, the BF provided strong evidence for a null effect. In the case of Trueblood et al. (2015), RST_UW_ indicated again a strong similarity effect, but according to RST_EW_, the BF provided equal support for a null and a similarity effect in Trueblood et al. (2015). Finally, in the case of Trueblood et al. (2014), the RST_UW_ measure pointed to equal support for a similarity and a null effect, but with the accurate RST_EW_, strong support for a null effect was found. For the remaining two studies (i.e., Liew et al., 2016; Berkowitsch et al., 2014), the BFs provided similar qualitative conclusions for the similarity effect.

For the compromise effect, the study of Berkowitsch et al. (2014) provided equal evidence for the absence and the presence of an effect when relying on RST_UW_ and examining its HDI (cf. Table 2), but when using the accurate RST_EW_, the BF indicated a strong effect. In all other studies, regardless of the RST measure used, the BF provided moderate to strong support for no compromise effect having occurred.

Furthermore, both RST measures provided substantial and consistent evidence for the attraction effect in the Berkowitsch et al. (2014) and Liew et al. (2016) studies when relying on the BFs. In the study by Cataldo and Cohen (2019), both RST measures provided strong to moderate evidence against an attraction effect. In contrast, the two RST measures led to different conclusions in the study by Trueblood et al. (2014) and Trueblood et al. (2015). In Trueblood et al. (2014), the RST_UW_ measure provided evidence for both a null and an attraction effect, but when relying on the accurate RST_EW_ measure, strong evidence for an attraction effect was observed. In Trueblood et al. (2015), on the other hand, the RST_UW_ measure indicated equal evidence for either the null or the attraction effect, whereas according to the accurate RST_EW_ measure, there was moderate evidence for a null effect. Overall, the conclusions of the BFs of the two measures differed in six of 15 cases, with most cases involving the similarity (three cases) and compromise (one case) effects and two cases concerning the attraction effect.

In general, the BF analysis of the RST measures was more conservative against the alternative hypothesis, establishing six corrections in total in contrast to the HDI analysis (cf. Table 2), which established four. When considering the accurate RST_EW_ measure, the cases in which the HDIs of the RST_EW_ measure established an effect with a lower boundary close to 0.50 were usually considered evidence for the null effect (i.e., compromise effect in Liew et al. 2016 and attraction effect in Trueblood et al., 2015) or equal evidence for and against the existence of an effect (i.e., attraction effect in Trueblood et al., 2015). For the rest of the RST_EW_ cases, the results of the HDI and BF analyses coincided. It should be noted that HDIs are based on the estimated alternative-hypothesis model, which is assumed to be true, and HDIs do not represent an explicit model comparison test, as is the case with the BF approach. Thus, HDIs can, in some instances, be more biased in rejecting the null hypothesis compared to BFs (cf. Wagenmakers et al., 2019). However, this aspect goes beyond the point of the present study.

**Table 6:** *B**F*_10_ of the hierarchical mean RST measure by study, method (unequal vs. equal weights), and context effect

Study	Attraction	Compromise	Similarity
Unequal weights			
Berkowitsch et al. (2014)	1000	0.535	0.046
Cataldo and Cohen (2019)	0.01	0.042	> 1000
Liew et al. (2016)	> 1000	0.017	0.057
Trueblood et al. (2014)	2.509	0.157	0.366
Trueblood et al. (2015)	2.791	0.03	87.852
Equal weights			
Berkowitsch et al. (2014)	> 1000	11.558	0.101
Cataldo and Cohen (2019)	0.207	0.029	0.009
Liew et al. (2016)	> 1000	0.177	0.034
Trueblood et al. (2014)	26.923	0.105	0.026
Trueblood et al. (2015)	0.309	0.045	0.345

Values are rounded to the third decimal. BF_10_ = Bayes factor in favor of the alternative hypothesis; RST = relative choice share of the targe

## Appendix D: Regularity and absolute choice share of target (AST) and competitor (ASC)

Assume two core options *A* and *B*, embedded in a choice pair {*A*,*B*} and in two triplet sets {*A*,*B*,*D*_1_} and {*A*,*B*,*D*_2_} where the decoy (*D*_1_) targets *A* in Set 1 and the decoy (*D*_2_) targets *B* in Set 2. From regularity it follows that the following inequalities hold:

D.1 $$P(A|\{A, B\}) \geq P(A|\{A, B, D_{1}\}),$$

D.2 $$P(A|\{A, B\}) \geq P(A|\{A, B, D_{2}\}),$$

D.3 $$P(B|\{A, B\}) \geq P(B|\{A, B, D_{1}\}),$$

D.4 $$P(B|\{A, B\}) \geq P(B|\{A, B, D_{2}\}).$$

Moreover, from probability theory, we know that *P*(*A*|{*A*,*B*}) + *P*(*B*|{*A*,*B*}) = 1. Consequently, the following propositions should also hold:

D.5 $$P(A|\{A, B, D_{1}\}) + P(B|\{A, B, D_{1}\}) \leq 1,$$

D.6 $$P(A|\{A, B, D_{2}\}) + P(B|\{A, B, D_{2}\}) \leq 1,$$

D.7 $$P(A|\{A, B, D_{1}\}) + P(B|\{A, B, D_{2}\}) \leq 1,$$

D.8 $$P(A|\{A, B, D_{2}\}) + P(B|\{A, B, D_{1}\}) \leq 1.$$

Since inequalities Eqs. D.5 and D.6 are always true because of the definition of the multinomial distribution, inequalities Eqs. D.7 and D.8 should be used as a test for regularity. However, to keep the test similar to RST, we reformulate inequalities Eqs. D.7 and D.8, respectively, as follows:

D.9 $$0.5*(P(A|\{A, B, D_{1}\}) + P(B|\{A, B, D_{2}\})) \leq 0.5,$$

D.10 $$0.5*(P(B|\{A, B, D_{1}\}) + P(A|\{A, B, D_{2}\})) \leq 0.5.$$

Given that *A* is the target of the attraction effect in Context Set 1 and *B* is the target in Context Set 2, inequalities Eqs. D.9 and D.10 correspond to the AST and ASC, respectively.

## Footnotes

1 We obtained similar simulation results with a probit implementation of the model.

2 Importantly, the RST_EW_ measure captures the correct underlying effect both when the alternative and when the null hypothesis is true (see supplementary materials).

3 The reduced degrees of freedom in the *t* tests of RST_EW_ in the compromise and similarity effects of (Trueblood et al., 2014) in Table 3 are caused by a participant whose RST could not be calculated because of zero target/competitor observations in one choice set.

4 Note that if *n*_d,C1_ = 0 and *n*_d,C2_ = 0, then AST reduces to RST_EW_.

5 We thank an anonymous reviewer for pointing this out to us.

## References

[CR1] Aczel B, Palfi B, Szollosi A, Kovacs M, Szaszi B, Szecsi P, Zrubka M, Gronau QF, van den Bergh D, Wagenmakers E-J (2018). Quantifying support for the null hypothesis in psychology: An empirical investigation. Advances in Methods and Practices in Psychological Science.

[CR2] Amir O, Levav J (2008). Choice construction versus preference construction: The instability of preferences learned in context. Journal of Marketing Research.

[CR3] Berkowitsch NAJ, Scheibehenne B, Rieskamp J (2014). Rigorously testing multialternative decision field theory against random utility models. Journal of Experimental Psychology: General.

[CR4] Bettman JR, Luce MF, Payne JW (1998). Constructive consumer choice processes. Journal of Consumer Research.

[CR5] Bhatia S (2013). Associations and the accumulation of preference. Psychological Review.

[CR6] Busemeyer JR, Barkan R, Mehta S, Chaturvedi A (2007). Context effects and models of preferential choice: implications for consumer behavior. Marketing Theory.

[CR7] Busemeyer JR, Gluth S, Rieskamp J, Turner BM (2019). Cognitive and neural bases of multi-attribute, multi-alternative, value-based decisions. Trends in Cognitive Sciences.

[CR8] Carpenter B, Gelman A, Hoffman MD, Lee D, Goodrich B, Betancourt M, Brubaker M, Guo J, Li P, Riddell A (2017). Stan: A probabilistic programming language. Journal of Statistical Software.

[CR9] Cataldo AM, Cohen AL (2019). The comparison process as an account of variation in the attraction, compromise, and similarity effects. Psychonomic Bulletin & Review.

[CR10] Choplin JM, Hummel JE (2005). Comparison-induced decoy effects. Memory & Cognition.

[CR11] Cumming G (2014). The New statistics: Why and how. Psychological Science.

[CR12] Debreu G (1960). Review of individual choice behavior: A theoretical analysis. The American Economic Review.

[CR13] Dhar R, Simonson I (2003). The Effect of Forced Choice on Choice. Journal of Marketing Research.

[CR14] Dienes Z (2016). How Bayes factors change scientific practice. Bayes Factors for Testing Hypotheses in Psychological Research: Practical Relevance and New Developments. Journal of Mathematical Psychology.

[CR15] Dumbalska T, Li V, Tsetsos K, Summerfield C (2020). A map of decoy influence in human multialternative choice. Proceedings of the National Academy of Sciences.

[CR16] Evans NJ, Holmes WR, Trueblood JS (2019). Response-time data provide critical constraints on dynamic models of multi-alternative, multi-attribute choice. Psychonomic Bulletin & Review.

[CR17] Evans NJ, Holmes W, Dasari A, Trueblood J (2021). The impact of presentation order on attraction and repulsion effects in decision-making. Decision.

[CR18] Farmer GD, Warren PA, El-Deredy W, Howes A (2017). The effect of expected value on attraction effect preference reversals. Journal of Behavioral Decision Making.

[CR19] Gigerenzer G, Marewski JN (2015). Surrogate science: The idol of a universal method for scientific inference. Journal of Management.

[CR20] Gronau QF, Sarafoglou A, Matzke D, Ly A, Boehm U, Marsman M, Leslie DS, Forster JJ, Wagenmakers Eric-Jan, Steingroever H (2017). A tutorial on bridge sampling. Journal of Mathematical Psychology.

[CR21] Gronau QF, Singmann H, Wagenmakers E-J (2020). Bridgesampling: An r package for estimating normalizing constants. Journal of Statistical Software.

[CR22] Heath TB, Chatterjee S (1995). Asymmetric decoy effects on lower-quality versus higher-quality brands: meta-analytic and experimental evidence. Journal of Consumer Research.

[CR23] Heck DW (2019). A caveat on the SavageDickey density ratio: The case of computing Bayes factors for regression parameters. British Journal of Mathematical and Statistical Psychology.

[CR24] Howes A, Warren PA, Farmer G, El-Deredy W, Lewis RL (2016). Why contextual preference reversals maximize expected value. Psychological Review.

[CR25] Huber J, Payne JW, Puto C (1982). Adding asymmetrically dominated alternatives: Violations of regularity and the similarity hypothesis. Journal of Consumer Research.

[CR26] Hutchinson JW, Kamakura WA, Lynch JG (2000). Unobserved heterogeneity as an alternative explanation for “reversal” effects in behavioral research. Journal of Consumer Research.

[CR27] Ioannidis JPA (2005). Why most published research findings are false. PLOS Medicine.

[CR28] Jaeger TF (2008). Categorical data analysis: Away from ANOVAs (transformation or not) and towards logit mixed models. Special Issue: Emerging Data Analysis. Journal of Memory and Language.

[CR29] Kass RE, Raftery AE (1995). Bayes Factors. Journal of the American Statistical Association.

[CR30] Kruschke JK, Liddell TM (2018). The Bayesian new statistics: Hypothesis testing, estimation, meta-analysis, and power analysis from a Bayesian perspective. Psychonomic Bulletin & Review.

[CR31] Liew SX, Howe PDL, Little DR (2016). The appropriacy of averaging in the study of context effects. Psychonomic Bulletin & Review.

[CR32] Louie K, Khaw MW, Glimcher PW (2013). Normalization is a general neural mechanism for context-dependent decision making. Proceedings of the National Academy of Sciences.

[CR33] Luce RD (1959). Individual choice behavior: A theoretical analysis.

[CR34] Luce RD (1977). The choice axiom after twenty years. Journal of Mathematical Psychology.

[CR35] Malkoc SA, Hedgcock W, Hoeffler S (2013). Between a rock and a hard place: The failure of the attraction effect among unattractive alternatives. Journal of Consumer Psychology.

[CR36] Mishra S, Umesh UN, Stem DE (1993). Antecedents of the attraction effect: An information-processing approach. Journal of Marketing Research.

[CR37] Mohr PNC, Heekeren HR, Rieskamp J (2017). Attraction effect in risky choice can be explained by subjective distance between choice alternatives. Scientific Reports.

[CR38] Molloy MF, Galdo M, Bahg G, Liu Q, Turner BM (2019). Whats in a response time?: On the importance of response time measures in constraining models of context effects. Decision.

[CR39] Munafó MR, Nosek BA, Bishop DVM, Button KS, Chambers CD, Sert NPd, Simonsohn U, Wagenmakers E-J, Ware JJ, Ioannidis JPA (2017). A manifesto for reproducible science. Nature Human Behaviour.

[CR40] Neumann N, Bckenholt U, Sinha A (2016). A meta-analysis of extremeness aversion. Journal of Consumer Psychology.

[CR41] Noguchi T, Stewart N (2018). Multialternative decision by sampling: A model of decision making constrained by process data. Psychological Review.

[CR42] Nuzzo R (2014). Scientific method: Statistical errors. Nature News.

[CR43] O’Curry YPS, Pitts R (1995). The attraction effect and political choice in two elections. Journal of Consumer Psychology.

[CR44] Regenwetter M, Dana J, Davis-Stober CP (2011). Transitivity of preferences. Psychological Review.

[CR45] Rieskamp J, Busemeyer JR, Mellers BA (2006). Extending the bounds of rationality: evidence and theories of preferential choice. Journal of Economic Literature.

[CR46] Roe RM, Busemeyer JR, Townsend JT (2001). Multialternative decision field theory: a dynamic connectionist model of decision making. Psychological Review.

[CR47] Rumelhart DL, Greeno JG (1971). Similarity between stimuli: An experimental test of the Luce and Restle choice models. Journal of Mathematical Psychology.

[CR48] Simonson I (1989). Choice based on reasons: The case of attraction and compromise effects. Journal of Consumer Research.

[CR49] Simonson I, Tversky A (1992). Choice in context: Tradeoff contrast and extremeness aversion. Journal of Marketing Research.

[CR50] Soltani A, Martino BD, Camerer C (2012). A range-normalization model of context-dependent choice: A new model and evidence. PLOS Computational Biology.

[CR51] Spektor MS, Gluth S, Fontanesi L, Rieskamp Jrg (2019). How similarity between choice options affects decisions from experience: The accentuation-of-differences model. Psychological Review.

[CR52] Spektor MS, Kellen D, Hotaling JM (2018). When the good looks bad: An experimental exploration of the repulsion effect. Psychological Science.

[CR53] Trueblood JS (2012). Multialternative context effects obtained using an inference task. Psychonomic Bulletin & Review.

[CR54] Trueblood JS (2015). Reference point effects in riskless choice without loss aversion. Decision.

[CR55] Trueblood JS, Brown SD, Heathcote A (2014). The multiattribute linear ballistic accumulator model of context effects in multialternative choice. Psychological Review.

[CR56] Trueblood JS, Brown SD, Heathcote A (2015). The fragile nature of contextual preference reversals: Reply to Tsetsos, Chater, and Usher (2015). Psychological Review.

[CR57] Trueblood JS, Brown SD, Heathcote A, Busemeyer JR (2013). Not just for consumers: Context effects are fundamental to decision making. Psychological Science.

[CR58] Turner BM, Schley DR, Muller C, Tsetsos K (2018). Competing theories of multialternative, multiattribute preferential choice. Psychological Review.

[CR59] Tversky A (1972). Elimination by aspects: A theory of choice. Psychological Review.

[CR60] Tversky A, Russo J (1969). Substitutability and similarity in binary choices. Journal of Mathematical Psychology.

[CR61] Tversky A, Simonson I (1993). Context-Dependent Preferences. Management Science.

[CR62] Usher M, McClelland JL (2004). Loss Aversion and Inhibition in Dynamical Models of Multialternative Choice. Psychological Review.

[CR63] Wagenmakers E-J (2007). A practical solution to the pervasive problems of p values. Psychonomic Bulletin & Review.

[CR64] Wagenmakers E-J, Marsman M, Jamil T, Ly A, Verhagen J, Love J, Selker R, Gronau QF, mra M, Epskamp S, Matzke D, Rouder JN, Morey RD (2018). Bayesian inference for psychology. Part I: Theoretical advantages and practical ramifications. Psychonomic Bulletin & Review.

[CR65] Wagenmakers E-J, Wetzels R, Borsboom D, van der Maas HLJ, Kievit RA (2012). An agenda for purely confirmatory research. Perspectives on Psychological Science: A Journal of the Association for Psychological Science.

[CR66] Wedell DH (1991). Distinguishing among models of contextually induced preference reversals. Journal of Experimental Psychology: Learning, Memory, and Cognition.

[CR67] Wedell DH, Pettibone JC (1996). Using judgments to understand decoy effects in choice. Organizational Behavior and Human Decision Processes.

[CR68] Windschitl PD, Chambers JR (2004). The dud-alternative effect in likelihood judgment. Journal of Experimental Psychology: Learning, Memory, and Cognition.

[CR69] Wollschlaeger LM, Diederich A (2020). Similarity, attraction, and compromise effects: Original findings, recent empirical observations, and computational cognitive process models. The American Journal of Psychology.

[CR70] Gelman, A., Carlin, J. B., Stern, H. S., Duson, D. B., & Vehtari, A. (2013). Bayesian data analysis. Chapman and Hall/CRC.

[CR71] Hotaling, J., & Rieskamp, J. (2018). A quantitative test of computational models of multialternative context effects. Decision 6(201-222). 10.1037/dec0000096

[CR72] Lee, M. D., & Wagenmakers, E.-J. (2014). Bayesian cognitive modeling: A practical course. Cambridge University Press.

[CR73] Wagenmakers, E.-J., Lee, M., Rouder, J. N., & Morey, R. D. (2019). The principle of predictive irrelevance, or why intervals should not be used for model comparison featuring a point null hypothesis. 10.31234/osf.io/rqnu5

[CR74] Wollschläger, L. M., & Diederich, A. (2012). The 2N-ary choice tree model for N-alternative preferential choice. Frontiers in Psychology, 3. 10.3389/fpsyg.2012.00189 10.3389/fpsyg.2012.00189 PMC3378970 22723788

