# Supplementary material for: A robust Bayesian test for identifying context effects in multiattribute decision-making
Source: Psychon Bull Rev. 2022 Sep 27;30(2):498–515. doi: 10.3758/s13423-022-02157-2 (PMC10104952; doi:10.3758/s13423-022-02157-2)
Supplement: Supplementary file 1 — (PDF 13423˙2022˙2157˙MOESM1˙ESM.pdf) [file 13423_2022_2157_MOESM1_ESM.pdf]

Supplemmentary Materials of "A Robust Bayesian Test for Identifying Context Effects in Multiattribute Decision Making"

Simulation Results With Equal Sample Sizes Across Sets

Figure 1 shows the two relative choice share of the target (RST) methods, with unequal weights (RST_{UW}) and with equal weights (RST_{EW}) when the null hypothesis is true in the population and there is no sample-size difference across Sets 1 and 2, in which case the two methods are mathematically equivalent. As expected, the two methods agree, almost always supporting the null hypothesis with at least moderate evidence.

Figure 1

Results of a Bayesian Simulation With No Sample-Size Difference Between the Two Choice Sets

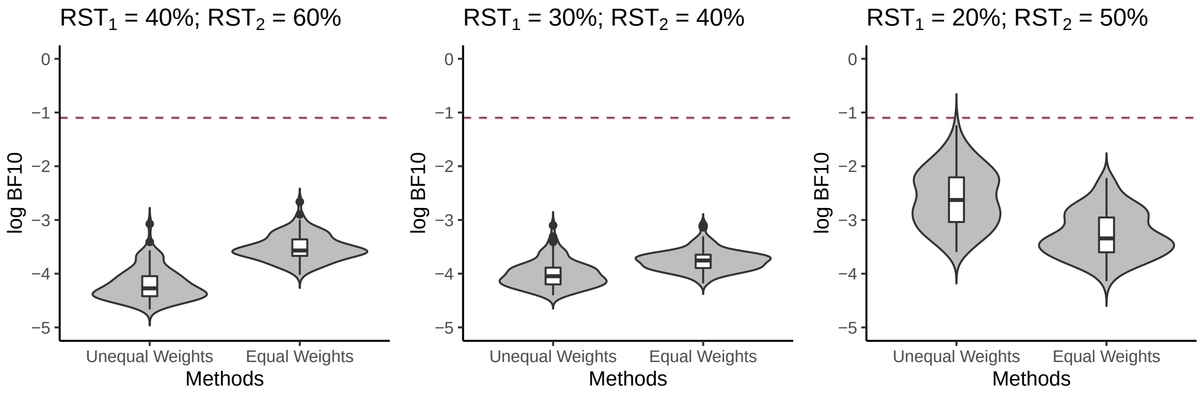

Note. Violin and box plots of log Bayes factor(BF) distributions from RST_{UW} and RST_{EW} are presented (BF_{10} indicates strength of evidence for the alternative hypothesis). The maroon dashed line indicates the BF = 1/3 threshold. In all scenarios, the null hypothesis is true in the population. RST = Relative choice share of the target; EW = equal weights; UW = unequal weights.

Simulation Results From the Frequentist Test

The results of the simulation of the frequentist test were similar to the those of the Bayesian test (see Figure 2). Generally, the *p* values from RST_{UW} were susceptible to changes in sample-size differences across sets. Specifically, when the null hypothesis was true in the population, *t* tests based on the RST_{UW} rejected the null more frequently with increasing set sample-size differences. This became more extreme when the RST of each set got closer to 0 or 1 (because of a difference in variance levels across the *p* parameter of the binomial distribution). In contrast, *t* tests of RST_{EW} did not get more significant with increasing sample-size difference and they stayed at the prespecified 5% false positive rate (α level). Note that in RST_{EW}, when the null hypothesis was true, *p* values did not have a certain direction but fluctuated uniformly between 0 and 1. This is a known problematic property of *p* values (Wagenmakers, 2007): The *p* values cannot differentiate between the reasons for not rejecting the null (i.e., because of ambiguous data or because the null hypothesis is indeed true).

When the alternative hypothesis was true in the population, *p* values based on RST_{EW} almost always rejected the null hypothesis irrespective of sample-size differences. However, this was not the case for RST_{UW}: When the set with RST = 70% had more observations than the other with RST = 50%, then the test rejected the alternative hypothesis, but when the set with RST = 50% had more observations than the other, then with increasing sample-size difference, *p* values did not reject the null hypothesis as much as before. This happens because RST_{UW} is a weighted average and shifts the average RST measure toward the set with the largest sample size, a pattern we also observed in the Bayesian measure of RST_{UW}. Moreover, even when the effect size is very small (in the scenario of $RST_1 = 0.50$ and $RST_2 = .60$ with an average RST of .55), most *p* values correctly rejected the null hypothesis in contrast to BFs, only a small proportion of which were in favor of the alternative hypothesis. This is a property of *p* values that has been called "violently biased" against the null hypothesis (Edwards, 1965, p. 400; for a review, see Wagenmakers et al.,

2018; Wetzels et al., 2011). BFs more conservatively reject the null (or the alternative) hypothesis. Though this property of p values appears advantageous to BFs in the results of our simulation, in the real world where the underlying true parameters are not known in advance, the conservativeness of BFs can protect against easily adopting positive results that rely on weak samples.

Figure 2

Results of Frequentist Simulation

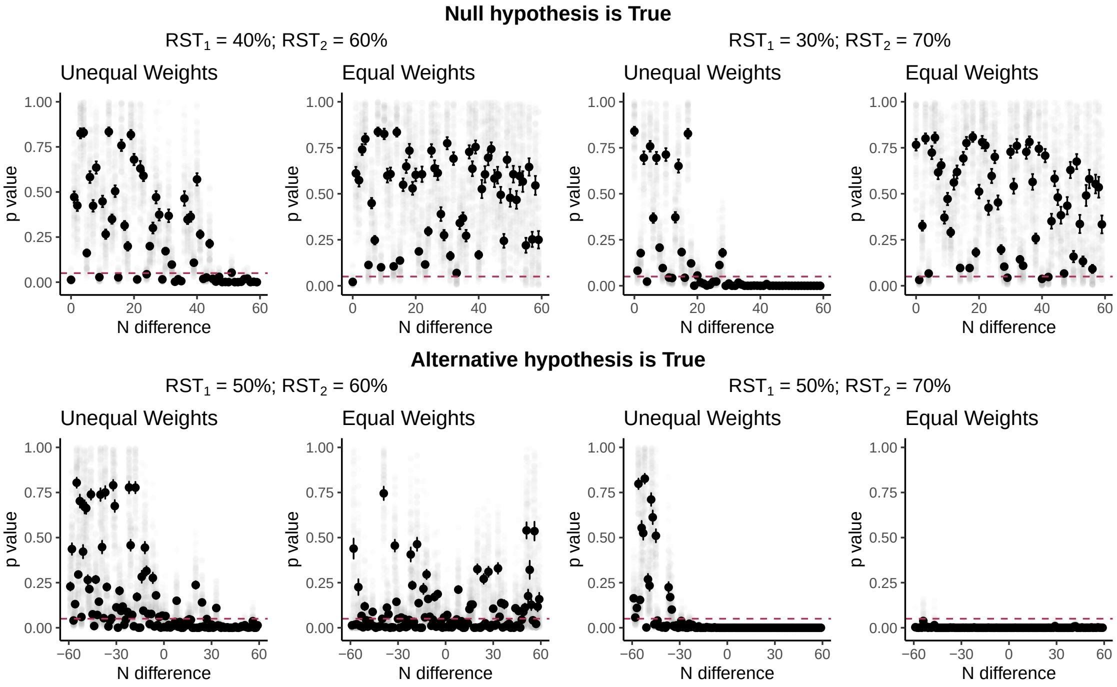

Note. The p values from RST_{UW} and RST_{EW} are presented. Black dots indicate means per unit N difference; gray dots indicate raw p values. Means are given with 95% confidence intervals. The maroon dashed line indicates the $p = .05$ threshold. EW = Equal weights; RST = relative choice share of the target; UW = unequal weights.

Direction of Posterior RST_{EW}

Figure 3

Percentage of the Direction of RST_{EW} Based on the Highest Density Intervals of the Posterior Distribution

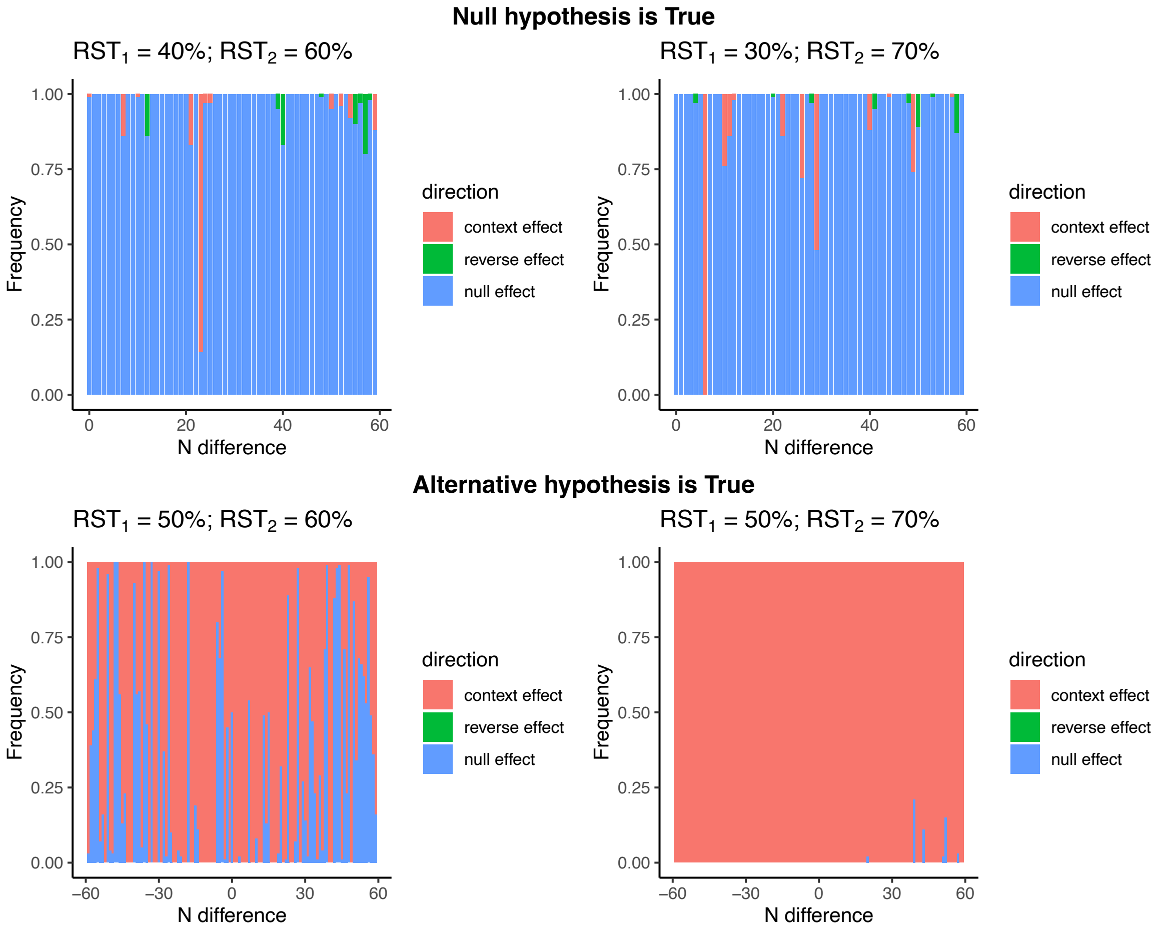

Note. Different scenarios correspond to the simulation study found in the paper. EW = equal weights; RST_1 = relative choice share of the target in Set 1; RST_2 = relative choice share of the target in Set 2.

List of Journal Articles Relevant to Context Effects in Four Psychological Journals

Table 1

Output of the Bibliographic Search of Four Major Psychological Journals

Reference	Journal
Spektor et al., 2019	Psych Rev
Bhui & Gershman, 2018	Psych Rev
Noguchi & Stewart, 2018	Psych Rev
Turner et al., 2018	Psych Rev
Howes et al., 2016	Psych Rev
Tsetsos et al., 2015	Psych Rev
Trueblood et al., 2015	Psych Rev
Trueblood et al., 2014	Psych Rev
Kintsch, 2014	Psych Rev
Bhatia, 2013	Psych Rev
Tsetsos et al., 2010	Psych Rev
Cataldo & Cohen, 2019	PBR
Liew et al., 2016	PBR
Trueblood, 2012	PBR
Spektor et al., 2018	Psych Science
Trueblood et al., 2013	Psych Science
Berkowitsch et al., 2014	JEPG

Note. The papers present research on context effects. Five of these papers met our inclusion criteria and were included in our reanalysis. Psych Rev = *Psychological Review*; PBR = *Psychonomic Bulletin Review*; Psych Science = *Psychological Science*; JEPG = *Journal of Experimental Psychology: General.*

Why $\mathbf{RST_{EW}}$ and $\mathbf{RST_{UW}}$ Agree on Correlations Among Context Effects

The $\mathrm{RST}_{\mathrm{EW}}$ and $\mathrm{RST}_{\mathrm{UW}}$ measures drew similar inferences about the existence of correlations among context effects because, according to the multivariate normal model, correlations of variables are not affected by changes in their marginal means. Therefore, although the $\mathrm{RST}_{\mathrm{EW}}$ and $\mathrm{RST}_{\mathrm{UW}}$ measures can disagree on the true location of the mean context effects, mean differences play no role in correlations of these effects. Only $\mathrm{RST}_{\mathrm{EW}}$ and $\mathrm{RST}_{\mathrm{UW}}$ estimating the variance of a context effect differently could make the measures draw different conclusions on correlation coefficients. This is because correlation is a standardized covariance measure, and the more the variance in one marginal distribution exceeds the other, the more covariation diminishes. Therefore, the fact that $\mathrm{RST}_{\mathrm{EW}}$ and $\mathrm{RST}_{\mathrm{UW}}$ made similar inferences on correlation coefficients among context effects led us to hypothesize that the two methods agreed on the underlying variance within each context effect.

To test this hypothesis, we used the mean posterior hierarchical parameters (i.e., μ and κ) per context effect, study, and measure to derive the variance of the beta distribution (from which all participant-based RSTs are sampled) according to the variance formula: $\frac{ab}{(a+b)^2+(a+b+1)}$, where a and b are the hierarchically derived beta parameters. The results are shown in Table 2 [where $a = \mu\kappa$ and $b = (1-\mu)\kappa$]. Generally, we observe that the estimated variance is comparable between the $\mathrm{RST}_{\mathrm{EW}}$ and $\mathrm{RST}_{\mathrm{UW}}$ measures. This confirms our intuition that the two RST measures draw similar inferences about the existence of correlations among context effects because they make similar inferences about their marginal variances.

Table 2

Variance Estimates of the Context-Effect Relative Choice Share of the Target (RST)

Effect	Method	Study	Variance
Attraction	Equal weights	Berkowitsch et al., 2014	0.230
Compromise	Equal weights	Berkowitsch et al., 2014	0.204
Similarity	Equal weights	Berkowitsch et al., 2014	0.245
Attraction	Unequal weights	Berkowitsch et al., 2014	0.233
Compromise	Unequal weights	Berkowitsch et al., 2014	0.209
Similarity	Unequal weights	Berkowitsch et al., 2014	0.234
Attraction	Equal weights	Cataldo & Cohen, 2019	0.080
Compromise	Equal weights	Cataldo & Cohen, 2019	0.102
Similarity	Equal weights	Cataldo & Cohen, 2019	0.088
Attraction	Unequal weights	Cataldo & Cohen, 2019	0.250
Compromise	Unequal weights	Cataldo & Cohen, 2019	0.250
Similarity	Unequal weights	Cataldo & Cohen, 2019	0.247
Attraction	Equal weights	Liew et al., 2016	0.199
Compromise	Equal weights	Liew et al., 2016	0.169
Similarity	Equal weights	Liew et al., 2016	0.178
Attraction	Unequal weights	Liew et al., 2016	0.219
Compromise	Unequal weights	Liew et al., 2016	0.180
Similarity	Unequal weights	Liew et al., 2016	0.191
Attraction	Equal weights	Trueblood et al., 2014	0.193
Compromise	Equal weights	Trueblood et al., 2014	0.107
Similarity	Equal weights	Trueblood et al., 2014	0.146
Attraction	Unequal weights	Trueblood et al., 2014	0.209
Compromise	Unequal weights	Trueblood et al., 2014	0.229
Similarity	Unequal weights	Trueblood et al., 2014	0.237
Attraction	Equal weights	Trueblood et al., 2015	0.233
Compromise	Equal weights	Trueblood et al., 2015	0.193
Similarity	Equal weights	Trueblood et al., 2015	0.206
Attraction	Unequal weights	Trueblood et al., 2015	0.247
Compromise	Unequal weights	Trueblood et al., 2015	0.246
Similarity	Unequal weights	Trueblood et al., 2015	0.242

Note. RST distributions (group-level beta distribution) are based on the mean posterior hierarchical parameters (μ and κ).

Reanalysis of Experiment 1 from Spektor et al. (2018)

Spektor et al. (2018) examined the conditions of the so-called repulsion effect, which describes situations in which the decoy does not boost the choice share of its similar but dominating option but rather the option that lies farther away in the multi-attribute space. Specifically, they showed attraction-effect stimuli to participants using perceptual stimuli in the gain domain (i.e., monetary gain for correct responses) and the loss domain (i.e., monetary loss for erroneous responses). The authors expected to find an attraction effect in the gain domain but a repulsion effect in the loss domain, reflecting the gain/loss framing effect (Tversky & Kahneman, 1981).

In their Experiment 1, Spektor et al. (2018) reported no difference in the RST across the two domains, and they further reported a repulsion effect in both the gain and the loss domains. The authors used the RST_{UW} in their calculation following the definition of Berkowitsch et al. (2014). However, as we showed in the main text, the absolute choice share of the target (AST) and the absolute choice share of the competitor (ASC) are the appropriate measures to evaluate violations of the regularity principle in attraction effect data (unlike the RST, which targets the independence from irrelevant alternatives principle). Following the statistical approach to hypothesis testing found in their study, we partly confirmed their results. Specifically, we found no AST difference across the gain and the loss domain, one-sided $t(60)$ = -0.412, p = .660, which agreed with the authors' conclusion that the attraction effect is not greater in the gain domain. However, unlike the authors, we did not find a repulsion effect according to the ASC (i.e., ASC > 50%) in either the gain, one-sided $t(30)$ = -0.65, p = .741, or the loss, one-sided $t(30)$ = -0.82, p = .791, domain. Taken together, the results from Experiment 1 do not provide evidence for violations of the regularity principle in the direction of a repulsion effect.

References

Berkowitsch, N. A. J., Scheibehenne, B., & Rieskamp, J. (2014). Rigorously testing multialternative decision field theory against random utility models. *Journal of Experimental Psychology: General*, *143*(3), 1331–1348. doi:https://doi.org/10.1037/a0035159

Bhatia, S. (2013). Associations and the accumulation of preference. *Psychological Review*, *120*(3), 522–543. doi:https://doi.org/10.1037/a0032457

Bhui, R., & Gershman, S. J. (2018). Decision by sampling implements efficient coding of psychoeconomic functions. *Psychological Review*, *125*(6), 985–1001. doi:https://doi.org/10.1037/rev0000123

Cataldo, A. M., & Cohen, A. L. (2019). The comparison process as an account of variation in the attraction, compromise, and similarity effects. *Psychonomic Bulletin & Review*, *26*(3), 934–942. doi:https://doi.org/10.3758/s13423-018-1531-9

Edwards, W. (1965). Tactical note on the relation between scientific and statistical hypotheses. *Psychological Bulletin*, *63*(6), 400–402. doi:https://doi.org/10.1037/h0021967

Howes, A., Warren, P. A., Farmer, G., El-Deredy, W., & Lewis, R. L. (2016). Why contextual preference reversals maximize expected value. *Psychological Review*, *123*(4), 368–391. doi:https://doi.org/10.1037/a0039996

Kintsch, W. (2014). Similarity as a function of semantic distance and amount of knowledge. *Psychological Review*, *121*(3), 559–561. doi:https://doi.org/10.1037/a0037017

Liew, S. X., Howe, P. D. L., & Little, D. R. (2016). The appropriacy of averaging in the study of context effects. *Psychonomic Bulletin & Review*, *23*(5), 1639–1646. doi:https://doi.org/10.3758/s13423-016-1032-7

Noguchi, T., & Stewart, N. (2018). Multialternative decision by sampling: A model of decision making constrained by process data. *Psychological Review*, *125*(4), 512–544. doi:https://doi.org/10.1037/rev0000102

Spektor, M. S., Gluth, S., Fontanesi, L., & Rieskamp, J. (2019). How similarity between choice options affects decisions from experience: The accentuation-of-differences model. *Psychological Review*, *126*(1), 52–88. doi:https://doi.org/10.1037/rev0000122

Spektor, M. S., Kellen, D., & Hotaling, J. M. (2018). When the good looks bad: An experimental exploration of the repulsion effect. *Psychological Science*, *29*(8), 1309–1320. doi:https://doi.org/10.1177/0956797618779041

Trueblood, J. S. (2012). Multialternative context effects obtained using an inference task. *Psychonomic Bulletin & Review*, *19*(5), 962–968. doi:https://doi.org/10.3758/s13423-012-0288-9

Trueblood, J. S., Brown, S. D., & Heathcote, A. (2014). The multiattribute linear ballistic accumulator model of context effects in multialternative choice. *Psychological Review*, *121*(2), 179–205. doi:https://doi.org/10.1037/a0036137

Trueblood, J. S., Brown, S. D., & Heathcote, A. (2015). The fragile nature of contextual preference reversals: Reply to Tsetsos, Chater, and Usher (2015). *Psychological Review*, *122*(4), 848–853. doi:https://doi.org/10.1037/a0039656

Trueblood, J. S., Brown, S. D., Heathcote, A., & Busemeyer, J. R. (2013). Not just for consumers: Context effects are fundamental to decision making. *Psychological Science*, *24*(6), 901–908. doi:https://doi.org/10.1177/0956797612464241

Tsetsos, K., Chater, N., & Usher, M. (2015). Examining the mechanisms underlying contextual preference reversal: Comment on Trueblood, Brown, and Heathcote (2014). *Psychological Review*, *122*(4), 838–847. doi:https://doi.org/10.1037/a0038953

Tsetsos, K., Usher, M., & Chater, N. (2010). Preference reversal in multiattribute choice. *Psychological Review*, *117*(4), 1275–1291. doi:https://doi.org/10.1037/a0020580

Turner, B. M., Schley, D. R., Muller, C., & Tsetsos, K. (2018). Competing theories of multialternative, multiattribute preferential choice. *Psychological Review*, *125*(3), 329–362. doi:https://doi.org/10.1037/rev0000089

Tversky, A., & Kahneman, D. (1981). The framing of decisions and the psychology of choice. *Science*, *211*(4481), 453–458. doi:https://doi.org/10.1126/science.7455683

Wagenmakers, E.-J. (2007). A practical solution to the pervasive problems of p values. *Psychonomic Bulletin & Review*, *14*(5), 779–804. doi:https://doi.org/10.3758/BF03194105

Wagenmakers, E.-J., Marsman, M., Jamil, T., Ly, A., Verhagen, J., Love, J., … Morey, R. D. (2018). Bayesian inference for psychology. Part I: Theoretical advantages and practical ramifications. *Psychonomic Bulletin & Review*, *25*(1), 35–57. doi:https://doi.org/10.3758/s13423-017-1343-3

Wetzels, R., Matzke, D., Lee, M. D., Rouder, J. N., Iverson, G. J., & Wagenmakers, E.-J. (2011). Statistical Evidence in Experimental Psychology: An Empirical Comparison Using 855 t Tests. *Perspectives on Psychological Science*, *6*(3), 291–298. doi:https://doi.org/10.1177/1745691611406923
